# RasGRP3 limits Toll-like receptor-triggered inflammatory response in macrophages by activating Rap1 small GTPase

**DOI:** 10.1038/ncomms5657

**Published:** 2014-08-14

**Authors:** Songqing Tang, Taoyong Chen, Zhou Yu, Xuhui Zhu, Mingjin Yang, Bin Xie, Nan Li, Xuetao Cao, Jianli Wang

**Affiliations:** 1 Institute of Immunology, Zhejiang University School of Medicine, Hangzhou 310058, China; 2 National Key Laboratory of Medical Immunology and Institute of Immunology, Second Military Medical University, 800 Xiangyin Road, Shanghai 200433, China; 3 National Key Laboratory of Medical Molecular Biology and Department of Immunology, Institute of Basic Medical Sciences, Chinese Academy of Medical Sciences, Beijing 100005, China; 4 These authors contributed equally to this work

## Abstract

Host immune cells can detect and destruct invading pathogens via pattern-recognition receptors. Small Rap GTPases act as conserved molecular switches coupling extracellular signals to various cellular responses, but their roles as regulators in Toll-like receptor (TLR) signalling have not been fully elucidated. Here we report that Ras guanine nucleotide-releasing protein 3 (RasGRP3), a guanine nucleotide-exchange factor activating Ras and Rap1, limits production of proinflammatory cytokines (especially IL-6) in macrophages by activating Rap1 on activation by low levels of TLR agonists. We demonstrate that RasGRP3, a dominant member of RasGRPs in macrophages, impairs TLR3/4/9-induced IL-6 production and relieves dextrane sulphate sodium-induced colitis and collagen-induced arthritis. In RasGRP3-deficient RAW264.7 cells obtained by CRISPR-Cas9 genome editing, TLR3/4/9-induced activation of Rap1 was inhibited while ERK1/2 activation was enhanced. Our study suggests that RasGRP3 limits inflammatory response by activating Rap1 on low-intensity pathogen infection, setting a threshold for preventing excessive inflammatory response.

## Abstract (web summary)

Toll like receptors (TLRs) couple microbial sensing to initiation of immune responses, which are essential for defense against pathogens but may cause immunopathology when activated excessively. Here the authors show that RasGRP3 sets a threshold of TLR activation to prevent immunopathology.

## Graphical abstract

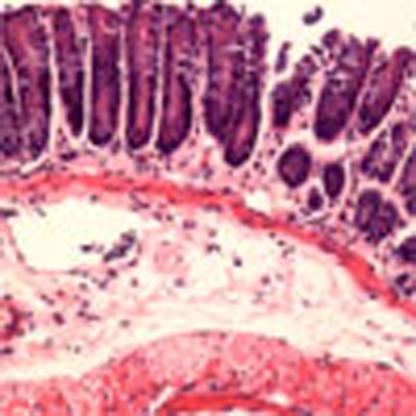

The host immune system is equipped with guarding receptors, namely pattern recognition receptors (PRRs), for detection and elimination of invading pathogens through activation of downstream signalling pathways and production of proinflammatory cytokines and type I interferons (IFNs, for example, IFNα/β)^1^. Toll-like receptors (TLRs) can detect components of pathogens and initiate the activation of mitogen-activated protein kinases (MAPKs) and nuclear factor-κB (NF-κB) pathways as well as the Tank-binding kinase (TBK)1-IFN regulatory factor (IRF)3 pathway through the adaptor molecules MyD88 (myeloid differentiation factor 88) and Toll-interleukin (IL)-1 receptor domain-containing adaptor molecule-1 (also known as TRIF)^1^. Retinoic acid-inducible gene 1 (*RIG-I*)-like receptors are mainly involved in sensing double-stranded RNA and activating IFNα/β production via the adaptor molecule mitochondrial antiviral-signalling protein (also known as IPS-1, VISA and CARDIF)^1^. The Nod-like receptors are involved in regulating the production of IL-1β via inflammasome activation^1^. Recently, cytoplasmic DNA sensors have been identified to recognize double-stranded DNA and activate IFNα/β primarily via stimulator of IFN genes (STING, also known as TMEM173, MITA and ERIS)^2^. Fine tuning of the PRRs’ signalling is critical for clearing pathogens and avoiding damages to host cells. Negative regulation of PRRs’ signalling, such as that mediated by short isoforms of signalling mediators, E3 ubiquitin ligases, deubiquitinating enzymes and phosphatases, has been explored^1^,^3^. However, due to the differential intensities of invasion during a natural course of infection, host cells need to distinguish the strength of infection and then respond properly to avoid damage from excessive response, the mechanisms for which have not been clearly understood.

Ras-related small GTPases, including Ras, Rab, Rho, Ran and ARF subfamilies of small GTPases, serve as critical molecular switches coupling extracellular signals to intracellular signalling pathways initiated by various receptors, such as growth factor receptors and G protein-coupled receptors^4^. By switching between GTP-bound ‘on’ and GDP-bound ‘off’ status, Ras-related small GTPases have been implicated in various aspects of cellular functions, such as adhesion, migration, proliferation, differentiation, protein transport and tumorigenesis^4^. Up to date, little is known about the roles of Ras-related small GTPases in innate immunity. Activation of phosphatidylinositide 3-kinase (PI3K)-Akt pathway by Rac1, a member of the Rac subfamily of the Rho small GTPases, is required for TLR2-induced NF-κB activation^5^. Constitutively active Ras promotes IL-1-induced p38 MAPK activation while Rap1a (Ras-proximity-1a), a member of the Rap subfamily of the Ras family of GTPases, antagonizes Ras, and dominant-negative Ras inhibits IL-1-induced NF-κB and AP-1 activation^6^,^7^. Our previous study revealed that dominant-negative Ras impaired CpG oligodeoxynucleotide (ODN)-induced TLR9 signalling and inhibited the production of tumour necrosis factor-α (TNFα)^8^. Our studies also showed that Rab small GTPases, including Rab7b and Rab10, are involved in regulation of TLR4 and TLR9 signalling pathway^9^,^10^,^11^. Rap1 is ubiquitously expressed in most tissues and mediates broad cellular activities^12^,^13^. The Rap1 signal plays an important role in controlling cell–cell and cell–matrix adhesion by regulating integrins and other adhesion molecules^12^,^13^,^14^. However, whether or not other small GTPases (for example, Rap1) are involved in TLR signalling may need further investigations.

Ras guanine nucleotide-releasing protein (RasGRP) is one type of GEF (GTP/GDP exchange factor) responsible for the activation of Ras and/or Rap^14^,^15^. Each protein contains a Ras exchange motif to interact with Ras and/or Rap, CDC25, EF hands for Ca^2+^ binding and C1 domain for diacylglycerol (DAG) binding^14^,^15^,^16^. RasGRPs are expressed in different leukocytes and regulate cell activation. RasGRP1 is predominantly expressed in T lymphocytes^16^,^17^,^18^,^19^. RasGRP1^−/−^ mice have a marked deficiency in the development of single positive thymocytes; T-cell receptor-mediated extracellular signal-regulated kinases (ERK1/2) activation is totally blocked in RasGRP1^−/−^ thymocytes and in a Jurkat cell line deficient in RasGRP1 protein^18^,^19^. RasGRP2 has been shown to be critical in platelet activation^20^,^21^. On the other hand, RasGRP3 is preferentially expressed in B cells and is required for optimal activation of ERK1/2 on B-cell receptor cross-linking^22^,^23^. RasGRP4 is highly expressed in mast cells and implicated in allergic responses^24^,^25^. A model of RasGRP-mediated regulation of Ras/ERK as a graded control of lymphocyte activation has been proposed^26^,^27^. However, whether RasGRPs play a role in graded activation of macrophages in response to innate stimuli still remains poorly understood.

In the current study, we investigated the roles of RasGRPs in macrophages activation on TLRs ligation. We find that RasGRP3 (a unique RasGRP member activating both Ras and Rap1)^28^, but not RasGRP1, 2 and 4, limits the production of proinflammatory cytokines in macrophages by activating Rap1 on sensing extremely low levels of TLR ligands. RasGRP3, by enhancing Rap1-GTP, may set a threshold for preventing excessive inflammatory response to low levels of infection.

## Results

### Macrophages preferentially express RasGRP3

**Figure 1:**
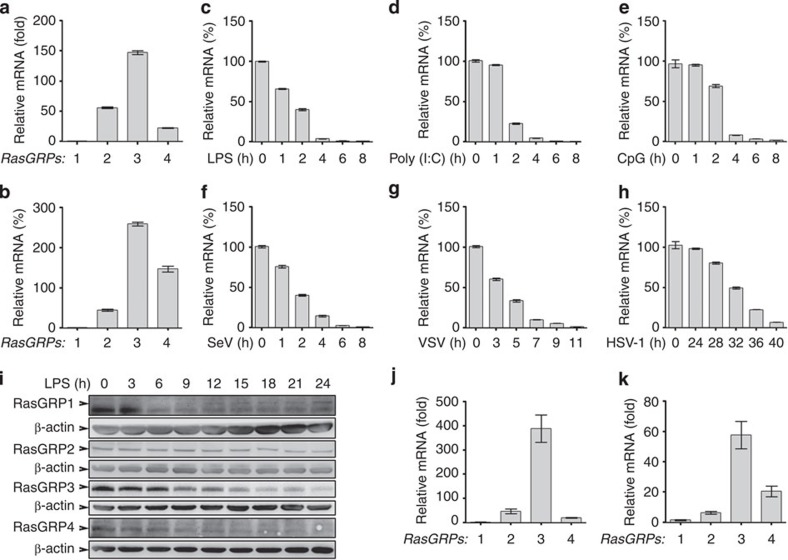
Dominant expression of RasGRP3 in macrophages. Peritoneal macrophages (**a**) and bone marrow-derived macrophages (**b**) from C57BL/6 wild-type mice were examined for *RasGRP1–4* expression by Q-PCR. (**c**–**h**) Peritoneal macrophages were treated with or without 100 ng ml^−1^ LPS (**c**), 10 μg ml^−1^ Poly (I:C) (**d**), 5 μM of CpG ODN (**e**), vesicular stomatitis virus (VSV; MOI=1; **f**), Sendi virus (SeV, MOI=1; **g**) or herpes simplex virus type 1 (HSV-1; MOI=1; **h**) as indicated. The mRNA levels of *RasGRP3* were examined by Q-PCR. (**i**) Peritoneal macrophages were treated with 100 ng ml^−1^ LPS as indicated, and the protein levels of RasGRP1-4 were examined by western blotting. Q-PCR assays of *RasGRP1-4* mRNA levels in PMA-treated (0.5 ng ml^−1^ for 12 h) THP-1 cells (**j**) and human MDM (**k**). Data are presented as mean±s.d. of triplicate samples and are representative of three independent experiments.

By examining the expression profiles of RasGRP1–4 in mouse peritoneal macrophages and mouse bone marrow-derived macrophages, we found that *RasGRP3* showed the highest expression level, sequentially followed by *RasGRP2* or *RasGRP4* (Fig. 1a,b). After lipopolysaccharide (LPS) treatment, *RasGRP3* was quickly and significantly downregulated (Fig. 1c). Similar results were obtained when peritoneal macrophages were stimulated by Poly (I:C), CpG ODN, vesicular stomatitis virus, Sendai virus or herpes simplex virus type 1 (Fig. 1d–h). In addition, we found that *RasGRP2* and *RasGRP4* were downregulated while RasGRP1 was upregulated by LPS, Poly (I:C) or CpG ODN (Supplementary Fig. 1).

At the protein level, we found that RasGRP1 and RasGRP4 were low/negative (long exposure time), while levels of RasGRP2 and RasGRP3 were high (short exposure time) in peritoneal macrophages (Fig. 1i). After LPS treatment, the levels of RasGRP2/3/4 were all decreased while RasGRP1 was induced.

We also examined the expression profiles of RasGRP1–4 in human THP-1 monocytic cells treated with phorbol-12-myristate-13-acetate (PMA) and human peripheral blood mononuclear cell-derived macrophages (monocyte-derived macrophages, MDMs). We found that *RasGRP3* was also the predominant RasGRP member in human macrophages (Fig. 1j,k). After LPS, Poly (I:C) or CpG ODN treatments, *RasGRP3*, similar to *RasGRP2* and *RasGRP4*, was rapidly downregulated while *RasGRP1* was upregulated by these TLR agonists (Supplementary Figs 2 and 3). These results indicate that RasGRP3 may be the major RasGRP member expressed by macrophages and may be involved in the innate activation of macrophages.

### RasGRP3 silence promotes proinflammatory cytokine production

**Figure 2:**
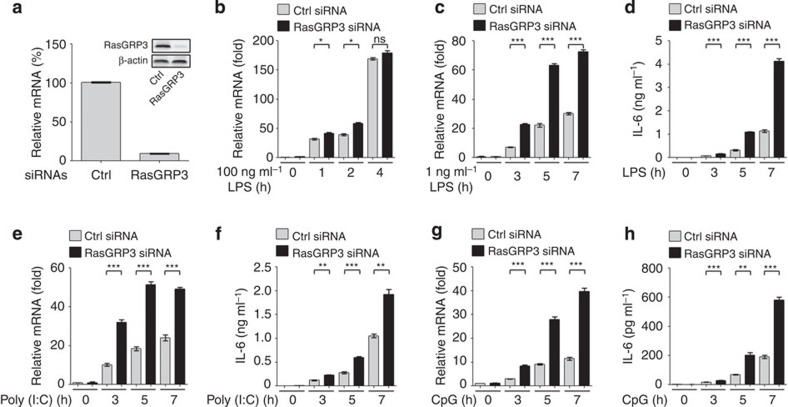
Knockdown of RasGRP3 promotes IL-6 production in mouse macrophages. (**a**) Peritoneal macrophages were transiently transfected with control (Ctrl) or RasGRP3-specific siRNAs for 48 h. mRNA levels of *RasGRP3* were examined by Q-PCR, and RasGRP3 protein was examined by western blotting (embedded image). (**b**–**h**) Cells in **a** were treated with 100 ng ml^−1^ LPS (**b**), 1 ng ml^−1^ LPS (**c**,**d**), 2.5 μg ml^−1^ Poly (I:C) (**e**,**f**) or 0.5 μM CpG ODN (**g**,**h**) for indicated times. Next, IL-6 levels were determined by Q-PCR (**b**,**c**,**e**,**g**) or ELISA (supernatants; **d**,**f**,**h**). Data are presented as mean±s.d. of triplicate samples and are representative of three independent experiments. ns, not significant; *P<0.05; **P<0.01; ***P<0.001 (analysis of variance).

Next we examined the roles of RasGRP1–4 in TLR response of macrophages after knockdown of individual RasGRPs. We found that RasGRP3 knockdown in mouse peritoneal macrophages (Fig. 2a) slightly but not significantly increased the expression of IL-6 when we stimulated the cells with 100 ng ml^−1^ LPS (the most widely used dose) (Fig. 2b). Considering the proposed characteristics of RasGRP (analogue) and SOS (digital) in activation of Ras/ERK in lymphocytes^26^,^27^, we analysed the function of RasGRP3 in peritoneal macrophages again by using low doses of stimulus. When stimulated with 1 ng ml^−1^ of LPS, peritoneal macrophages with RasGRP3 knockdown produced significantly higher levels of IL-6 (Fig. 2c,d). As compared with IL-6, the other inflammatory mediators, such as TNFα and inducible nitric oxide synthase (iNOS) but not IL-1β, were also significantly upregulated by RasGRP3 knockdown (Supplementary Fig. 4a–c). When stimulated with low doses of Poly (I:C) or CpG ODN, the peritoneal macrophages with RasGRP3 knockdown produced more IL-6 (Fig. 2e–h). These results indicate that RasGRP3 negatively regulates mouse macrophages responsiveness to TLR agonists.

However, the production of IL-6 was not affected when we silenced RasGRP1, RasGRP2 or RasGRP4 in peritoneal macrophage and stimulated the macrophages with 100 ng ml^−1^ LPS, 1 ng ml^−1^ LPS, 2.5 μg ml^−1^ Poly (I:C) or 0.5 μM CpG ODN (Supplementary Fig. 5). These data suggest that only RasGRP3 plays an important and negative role in regulating the activation of macrophages and production of proinflammatory cytokines on TLR activation.

**Figure 3:**
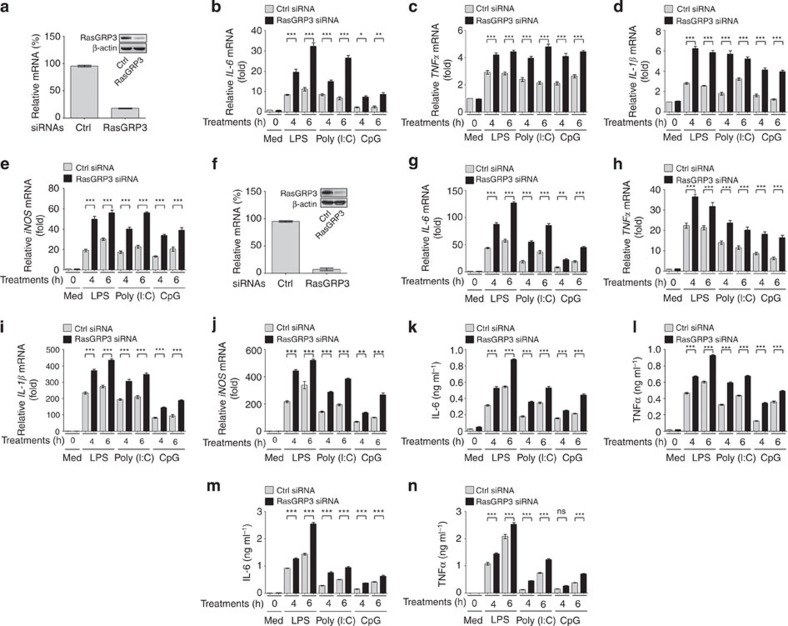
Knockdown of RasGRP3 promotes the production of proinflammatory cytokines in human macrophages. (**a**) THP-1 cells were treated with 0.5 ng ml^−1^ PMA for 12 h to differentiate into macrophages, and were transiently transfected with control (Ctrl) or RasGRP3-specific siRNAs for 48 h. mRNA levels of *RasGRP3* were examined by Q-PCR and RasGRP3 protein was examined by western blotting (embedded image). (**b**–**e**) Cells in **a** were treated with or without (Med) 1 ng ml^−1^ LPS, 2.5 μg ml^−1^ Poly (I:C) or 0.5 μM CpG ODN for indicated times. Next, *IL-6* (**b**), *TNF*α (**c**), *IL-1*β (**d**) and *iNOS* (**e**) mRNA levels were determined by Q-PCR. (**f**) Human peripheral mononuclear cells cultured with macrophage colony-stimulating factor (M-CSF) for 5 days (MDM) were transiently transfected with control (Ctrl) or RasGRP3-specific siRNAs for 48 h. mRNA levels of *RasGRP3* were examined by Q-PCR, and RasGRP3 protein was examined by western blotting (embedded image). (**g**–**j**) Cells in **f** were treated with or without (Med) 1 ng ml^−1^ LPS, 2.5 μg ml^−1^ Poly (I:C) or 0.5 μM CpG ODN for indicated times. Next, *IL-6* (**g**), *TNF*α (**h**), *IL-1*β (**i**) and *iNOS* (**j**) mRNA levels were determined by Q-PCR. (**k**–**n**) Culture supernatants derived from **b**,**c** or **g**,**h** were determined for IL-6 (**k**,**m**) and TNFα (**l**,**n**) levels by ELISA. Data are presented as mean±s.d. of triplicate samples and are representative of three independent experiments. ns, not significant; *P<0.05; **P<0.01; ***P<0.001 (analysis of variance).

In PMA-treated THP-1 cells and human MDMs, we also examined the effects of RasGRP3 knockdown on proinflammatory cytokines. We found that knockdown of RasGRP3 in both THP-1 cells and MDM significantly increased the production of IL-6, TNFα, IL-1β and iNOS at messenger RNA levels and/or protein levels after treatments with low doses of LPS, Poly (I:C) or CpG ODN (Fig. 3a–n). These data suggest that RasGRP3, similar to its roles in mouse macrophages, negatively regulates the TLR-triggered inflammatory responses in human macrophages.

To provide further evidence, we silenced the expression of RasGRP1, 2 and 4 in human THP-1 cells and MDM (Supplementary Fig. 6a,b). After treatments with the indicated TLR agonists, we found that knockdown of these RasGRPs did not affect the expression of IL-6 and TNFα (Supplementary Fig. 6c,d), further confirming that RasGRP3, but not RasGRP1, 2 or 4, is a negative regulator of TLR-triggered inflammatory responses in macrophages.

### RasGRP3 inhibits proinflammatory cytokine production

**Figure 4:**
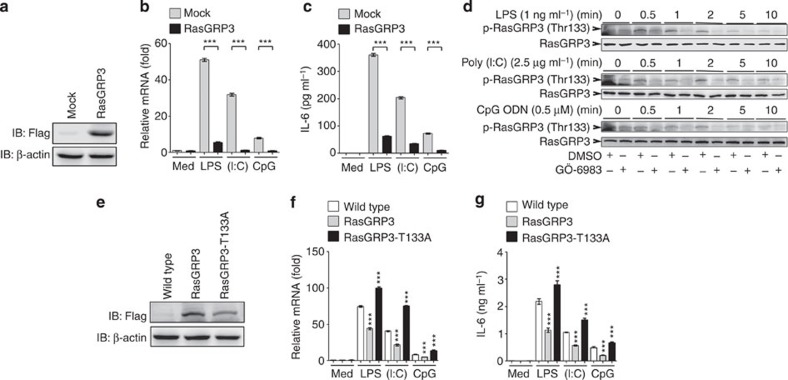
RasGRP3 inhibits IL-6 production in macrophages. (**a**–**c**) RAW264.7 cells stably transfected with Flag-tagged RasGRP3 were examined for RasGRP3 expression by western blotting (**a**), or treated with or without 1 ng ml^−1^ LPS, 2.5 μg ml^−1^ Poly (I:C) or 0.5 μM CpG ODN for 6 h (**b**,**c**). Next, *IL-6* mRNA levels and IL-6 proteins were examined by Q-PCR (**b**) and ELISA (**c**), respectively. (**d**) Bone marrow-derived macrophages were pretreated with dimethyl sulphoxide (DMSO) or GÖ-6983 (200 nM) for 30 min, and then stimulated with LPS, Poly (I:C) or CpG ODN as indicated. The phosphorylation of Thr133 in RasGRP3 was examined by using phospho-RasGRP3 (Thr133) Ab. (**e**–**g**) Peritoneal macrophages derived from wild-type mice, or RasGRP3 or RasGRP3-T133A transgenic mice were examined for RasGRP3 expression by western blotting (**e**), or treated with or without 1 ng ml^−1^ LPS, 2.5 μg ml^−1^ Poly (I:C) or 0.5 μM CpG ODN for 6 h (**f**,**g**). *IL-6* mRNA levels and the proteins were examined by Q-PCR (**f**) and ELISA (**g**), respectively. Data are presented as mean±s.d. of triplicate samples and are representative of three independent experiments. ***P<0.001 (analysis of variance).

To further verify the function of RasGRP3 in controlling the activation of macrophages, we examined the expression of IL-6 in RAW264.7 macrophages stably overexpressing RasGRP3 (Fig. 4a). We found that these cells produced less IL-6 than the control cells when stimulated with a low dose of LPS, Poly (I:C) or CpG ODN (Fig. 4b,c).

Previous studies have found that phosphorylation of Thr133 by protein kinase C is required for the activation of RasGRP3 in lymphocytes^23^,^29^,^30^. We examined the phosphorylation of Thr133 in RasGRP3 after TLR ligation, and found that the pan-protein kinase Cinhibitor GÖ-6983 could inhibit TLR-induced phosphorylation on Thr133 of RasGRP3 (Fig. 4d). To further characterize the roles of RasGRP3 in TLR signalling, we established transgenic mice overexpressing RasGRP3 and RasGRP3 with mutation of Thr133 to Ala (RasGRP3-T133A) (Supplementary Fig. 7 and Fig. 4e). In peritoneal macrophages derived from RasGRP3 and RasGRP3-T133A transgenic mice, we examined the production of IL-6 triggered by TLR3/4/9 ligations. We found that RasGRP3 transgenic macrophages showed a significant decrease of IL-6 production, while RasGRP3-T133A macrophages showed elevated IL-6 production (Fig. 4f,g). Therefore, our data convincingly demonstrate that RasGRP3 is a negative regulator of TLR signalling.

In human THP-1 cells, we also examined the effects of RasGRP3 overexpression on TLR-triggered IL-6 and TNFα production. We found that overexpression of wild-type RasGRP3 inhibited IL-6 and TNFα after LPS, Poly (I:C) or CpG ODN treatments (Supplementary Fig. 8a–g). Overexpression of RasGRP3-T133A promoted TLR3/4/9-induced IL-6 and TNFα production. In human colon cancer samples, we detected a mutation of RasGRP3, G558R (G1672 mutated to A; Supplementary Fig. 8h). When overexpressed in THP-1 cells, RasGRP3-G558R promoted IL-6 and TNFα production (Supplementary Fig. 8a–f). These data indicate that RasGRP3 negatively controls inflammatory cytokine production in human macrophages.

### RasGRP3 limits IL-6 production by activating Rap1

**Figure 5:**
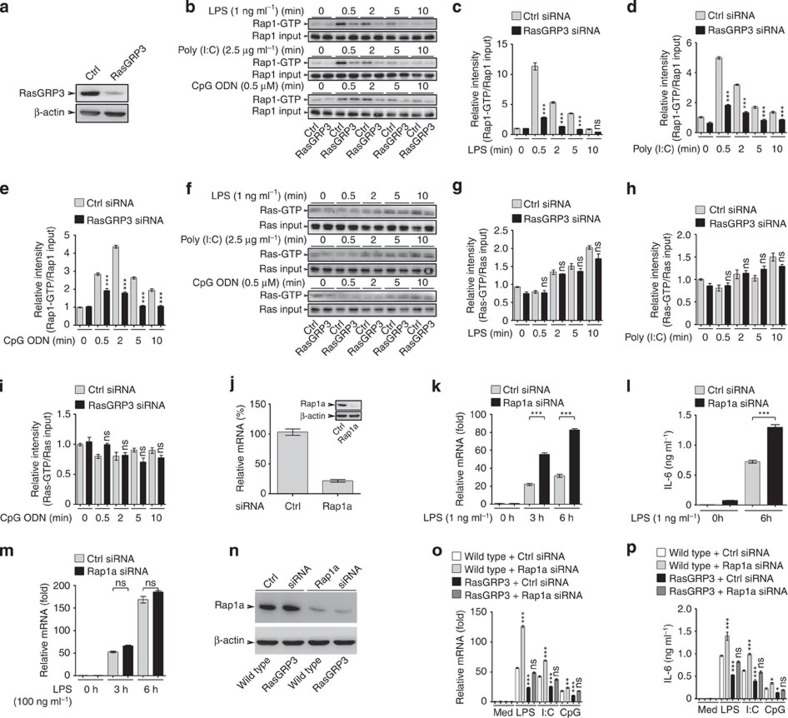
RasGRP3 inhibits IL-6 production via Rap1a activation. (**a**) Efficiency of RasGRP3 knockdown in peritoneal macrophages, as examined by western blotting. Levels of Rap1-GTP (**b**–**e**) and Ras-GTP (**f**–**i**) after LPS, Poly (I:C) or CpG ODN treatments as indicated. Extracts loaded with GDP or GTPγS were used as negative or positive controls for Rap1-GTP and Ras-GTP as recommended. The band intensity of indicated molecule were determined and calculated as GTP-bound Rap1 (**c**–**e**) or Ras (**g**–**i**) to Rap1 or Ras input. Data (**c**–**e**, **g**–**i**) are presented as mean±s.e. of three repeats. (**j**–**m**) Peritoneal macrophages were transfected with control siRNAs (Ctrl) or Rap1a-specific siRNAs (Rap1a) for 48 h. Levels of Rap1a were determined by Q-PCR and western blotting (**j**). Next, cells were treated with 1 ng ml^−1^ (**k**,**l**) or 100 ng ml^−1^ (**m**) LPS, and IL-6 levels were measured by Q-PCR (**k**,**m**) or ELISA (**l**). (**n**–**p**) Bone marrow-derived macrophages of wild-type (WT) mice or RasGRP3 transgenic mice were transfected with control siRNAs (Ctrl) or Rap1a-specific siRNAs (Rap1a) for 48 h. Next, Rap1a levels were examined by western blotting (**n**). After being treated with 1 ng ml^−1^ LPS for 6 h, the cells were examined for IL-6 levels by Q-PCR (**o**) and ELISA (**p**). Error bars in **k**–**m**,**o**,**p** indicate mean±s.d. of triplicate samples. All experiments were repeated for three times. ns, not significant; *P<0.05; **P<0.01; ***P<0.001 (analysis of variance, as indicated or as compared with wild-type cells plus control siRNAs).

Previous studies revealed that RasGRP3 is unique in that it can effectively activate small GTPases Ras and Rap1 (ref. 28). After knockdown of RasGRP3 in peritoneal macrophages (Fig. 5a), we found that the levels of Rap1-GTP (Fig. 5b–e), but not Ras-GTP (Fig. 5f–i), were markedly reduced after TLR ligands stimuli, indicating that Rap1 (but not Ras), activated by RasGRP3, may be responsible for early events in TLR signalling.

To confirm that Rap1 is a target of RasGRP3 in TLRs signalling in macrophages, we silenced the expression of Rap1a and Rap1b in peritoneal macrophages. We found that knockdown of Rap1a, but not Rap1b, could increase LPS-induced IL-6 production at 1 ng ml^−1^ but not 100 ng ml^−1^ (Fig. 5j–m), which were similar to the effects of RasGRP3 knockdown. These data indicate that RasGRP3 may regulate IL-6 production via activating Rap1a.

To obtain further evidence, we silenced Rap1a in bone marrow-derived macrophages from RasGRP3 transgenic mice (Fig. 5n) and examined IL-6 production after TLR agonist stimuli. We found that higher levels of IL-6 were detected in Rap1a knockdown group and silencing Rap1a could reverse the effects of RasGRP3 transgene on the production of TLR-triggered IL-6 (Fig. 5o,p).

### Knockdown of RasGRP3 promotes ERK1/2 activation

**Figure 6:**
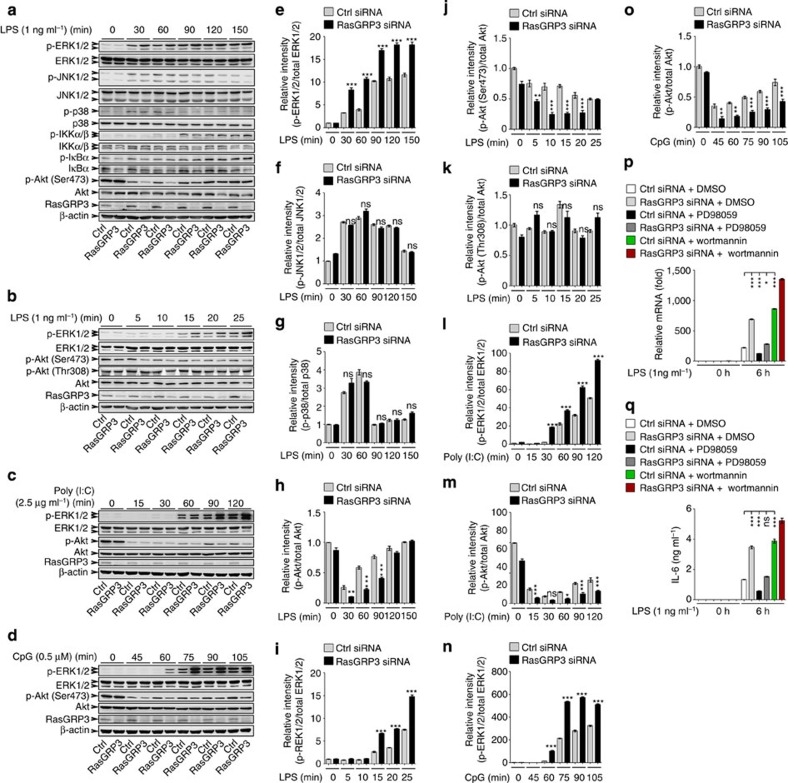
RasGRP3 knockdown accelerates dephosphorylation of Akt and activation of ERK1/2. (**a**–**d**) Peritoneal macrophages transfected with control siRNAs (Ctrl) or RasGRP3-specific siRNAs (RasGRP3) for 48 h were treated with indicated amounts of LPS, Poly (I:C) or CpG ODN for different times, and the levels of indicated molecules were examined by western blotting. (**e**–**o**) Results in **a**–**d** were quantified by determining the band intensity and calculated as phosphorylated signalling molecules to total corresponding molecules. Data were presented as mean±s.e. of three repeats. (**p**,**q**) Indicated peritoneal macrophages were pretreated with PD98059, Wortmannin or vehicle for 30 min, and then treated with 1 ng ml^−1^ LPS for 6 h. IL-6 levels were determined by Q-PCR (**p**) and ELISA (**q**). Error bars indicate mean±s.d. of triplicate samples. All experiments were repeated for three times. ns, not significant; *P<0.05; **P<0.01; ***P<0.001 (analysis of variance, as indicated or as compared with wild-type cells transfected with control siRNAs).

We then went to examine the effects of RasGRP3 knockdown on TLR-triggered signalling pathways (Fig. 6a–o). We found that silencing of RasGRP3 in peritoneal macrophages enhanced the activation of ERK1/2 stimulated by low doses of LPS, Poly (I:C) or CpG ODN (Fig. 6a–e,i,l,n). However, the activations of JNK1/2 and p38 MAPKs, as well as the IKKα/β-IκBα pathway were not significantly affected by RasGRP3 knockdown after LPS treatments (Fig. 6a,f,g).

As the PI3K-Akt pathway also plays crucial roles in regulating TLR response^1^,^31^, we examined the phosphorylation of Akt (Ser473). Unexpectedly, we found that the levels of phospho-Akt were initially decreased and then gradually restored in peritoneal macrophages after LPS, Poly (I:C) or CpG stimuli (Supplementary Fig. 9). Low doses of TLR agonists may have prolonged the duration of dephosphorylation process. In RasGRP3-silenced cells, the dephosphorylation of Akt was accelerated, thus maintaining a lower level of active Akt (Fig. 6a–d,h,j,m,o); moreover, the rephosphorylation of Akt since 30 min after LPS stimuli was also inhibited by RasGRP3 knockdown (Fig. 6a,h).

When the macrophages were pretreated with MEK/ERK inhibitor PD98059, the effects of RasGRP3 knockdown on IL-6 production were reversed (Fig. 6p,q), indicating that RasGRP3 knockdown-induced ERK1/2 activation may be responsible for the increased TLR4-triggered IL-6 production in macrophages. When the macrophages were pretreated with PI3K inhibitor Wortmannin, IL-6 production induced by LPS was also significantly increased, both in control and RasGRP3-silenced macrophages, which indicated that RasGRP3 knockdown-induced increase of IL-6 may be due to the accelerated Akt dephosphorylation.

### RasGRP3 limits IL-6 production *in vivo*

**Figure 7:**
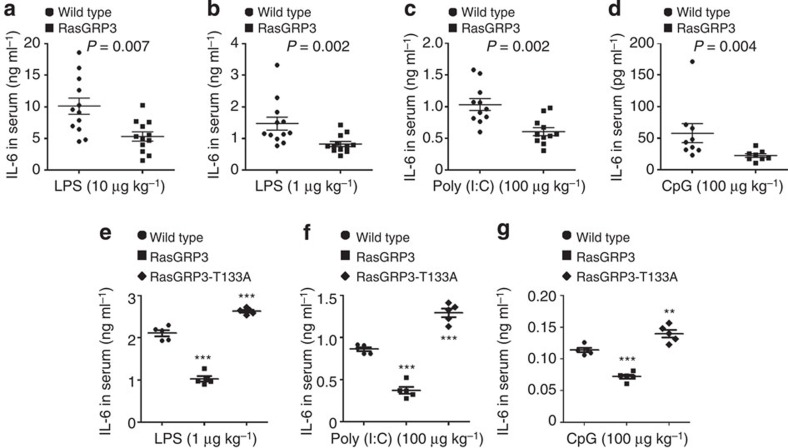
RasGRP3 inhibits TLR-triggered IL-6 production *in vivo*. (**a**–**d**) Wild-type mice, RasGRP3 or RasGRP3-T133A transgenic mice were injected with indicated amounts of LPS (**a**,**b**), Poly (I:C) (**c**) or CpG ODN (**d**) intraperitoneally. Two and a half hours later, IL-6 levels in serum were determined by ELISA. (**e**–**g**) Irradiated wild-type mice were intraperitoneally injected with wild-type, RasGRP3 or RasGRP3-T133A transgenic peritoneal macrophages 8 days after bone marrow transplantation. Two days after macrophages transfer, LPS (**e**), Poly (I:C) (**f**) or CpG ODN (**g**) were intraperitoneally injected. Two and a half hours later, IL-6 levels in serum were determined by ELISA. Data are presented as mean±s.d. (*n*=11 in **a**, *n*=12 in **b**, *n*=11 in **c**, *n*=9 in **d**, and *n*=5 in **e**,**f** and **g**). All experiments were repeated three times. **P<0.01; ***P<0.001 (Mann–Whitney *U*-test or analysis of variance, as compared with wild-type group).

To understand the role of RasGRP3 in the TLR-triggered innate immune response *in vivo*, we challenged RasGRP3 transgenic mice (Supplementary Fig. 7) with different doses of LPS intraperitoneally. When high doses of LPS were used (for example, 20 mg kg^−1^ or 200 μg kg^−1^), IL-6 levels in the serum of RasGRP3 transgenic mice and wild-type mice were comparable (Supplementary Fig. 10). However, when we used low doses of LPS (10 or 1 μg kg^−1^), IL-6 levels were significantly lower in the serum of RasGRP3 transgenic mice as compared with wild-type mice (Fig. 7a,b). Similarly, when we challenged the transgenic mice with low-dose of Poly (I:C) or CpG ODN, serum levels of IL-6 were also significantly decreased in RasGRP3 transgenic mice (Fig. 7c,d). We have also examined the levels of TNFα in the serum. However, we found that TNFα was undetectable in the serum of mice treated with low doses of TLR agonists. These data may indicate that RasGRP3 acts as a negative regulator of TLR innate response to low levels of agonists *in vivo*.

To further illustrate the contribution of RasGRP3 to IL-6 production in macrophages, we transferred wild-type or RasGRP3 transgenic macrophages to irradiated wild-type mice. After peritoneal administration of low doses of LPS, Poly (I:C) or CpG ODN, we found that IL-6 levels of mice reconstituted with RasGRP3 transgenic macrophages were significantly decreased while that of mice reconstituted with RasGRP3-T133A transgenic macrophages were significantly increased (Fig. 7e–g). These data suggest that RasGRP3-mediated inhibition of IL-6 production in macrophages is an intrinsic mechanism to limit inflammatory responses during infectious disease.

### RasGRP3 suppresses dextrane sulphate sodium-induced colitis

**Figure 8:**
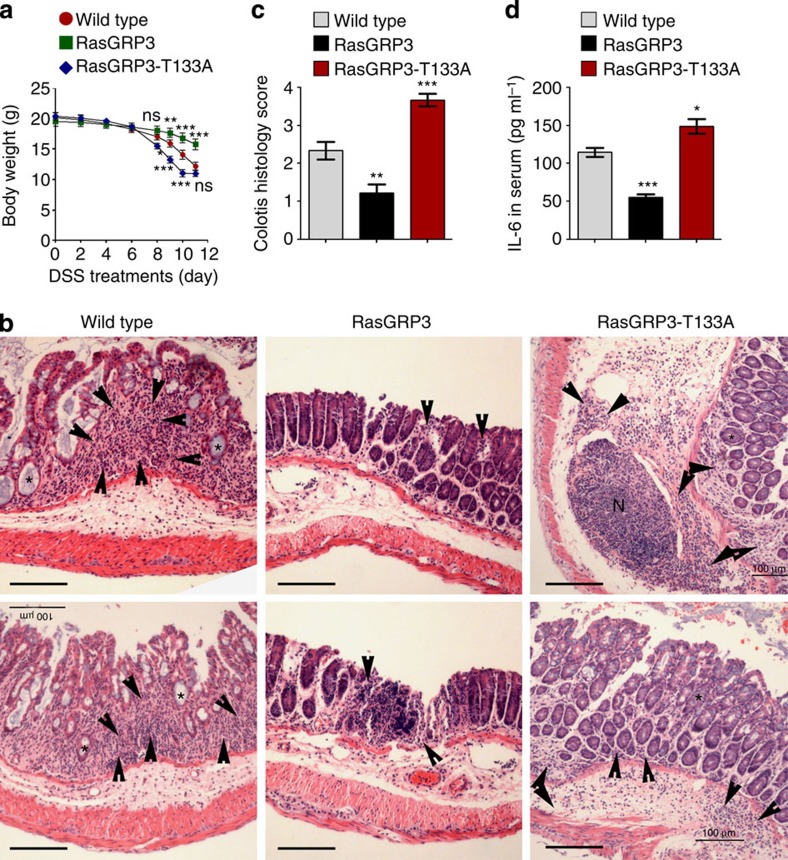
RasGRP3 inhibits the inflammation of DSS-induced colitis. Irradiated wild-type mice were intraperitoneally injected with wild-type, RasGRP3 or RasGRP3-T133A transgenic peritoneal macrophages 8 days after bone marrow transplantation. Two days after macrophages transfer, mice were fed with 2.5% DSS in water for 7 days. Body weights were determined on indicated days (**a**, *n*=8 per group). On day 10 after DSS treatments, the colons were collected and examined by H&E staining (**b**), the inflammation of colitis were evaluated by a pathologist (**c**, *n*=5 per group) and IL-6 levels in serum were measured by ELISA (**d**, *n*=5 per group). In **b**, infiltration of inflammatory cells was indicated with arrowheads, crypt abscesses were indicated with asterisks and large lymphoid nodules (N) were also illustrated. Note the differential distribution (scattered versus universal and mucous versus submucous) of infiltrated inflammatory cells. Results in **a**,**c**,**d** were presented as mean±s.d. All experiments were repeated three times. ns, not significant; *P<0.05; **P<0.01; ***P<0.001 (analysis of variance, as compared with wild-type groups). Scale bars, 100 μm.

Our data have suggested that RasGRP3 negatively regulates TLR-induced inflammatory response both *in vitro* and *in vivo*. We then went further to examine whether RasGRP3 was involved in the pathogenesis of inflammatory diseases. Dextrane sulphate sodium (DSS)-induced colitis is a typical inflammatory model to investigate the development and control of inflammatory bowel diseases (IBDs). To demonstrate the roles of RasGRP3-mediated regulation of macrophage responses in DSS-induced colitis, we transferred the RasGRP3 transgenic macrophages into irradiated wild-type mice and administered DSS. We found that the body weight of mice reconstituted with RasGRP3 transgenic macrophages showed slower decrease, as compared with that of mice reconstituted with wild-type macrophages (Fig. 8a). On day 10 after DSS treatments, colons were collected and examined by haematoxylin and eosin (H&E) staining. We found that colons derived from the RasGRP3 group demonstrated more mild colitis than those derived from wild-type group while colons derived from RasGRP3-T133A transgenic mice demonstrated the most severe colitis (Fig. 8b,c). In the serum, IL-6 levels were lower in RasGRP3 group, as compared with that in wild-type group (Fig. 8d). Levels of TNFα were also examined. However, TNFα levels in serum on day 10 of DSS treatments were undetectable by enzyme-linked immunosorbent assay (ELISA). These data indicated that RasGRP3 suppresses the development of inflammatory diseases such as IBD.

### RasGRP3 suppresses collagen-induced arthritis

Our data have suggested that RasGRP3 negatively regulates IL-6 production on TLR3/4/9 activation both *in vitro* and *in vivo*. However, the physiological or pathological significance of these observations has not been elucidated. Disease models have shown that IL-6 can induce local chemokine production and promote local inflammation during the development of rheumatoid arthritis (RA) and multiple sclerosis^32^,^33^. Many studies have shown that IL-6 plays an important role in RA^32^,^33^. Therefore, we tested whether RasGRP3 could affect the development of collagen-induced arthritis (CIA).

**Figure 9:**
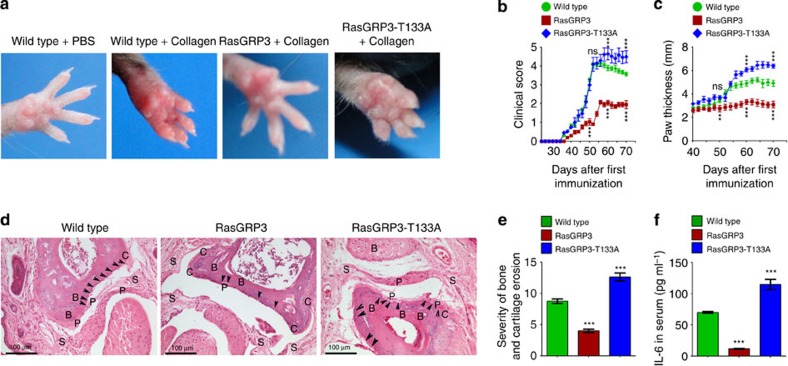
RasGRP3 regresses the development of CIA. (**a**) Wild-type mice, or RasGRP3 or RasGRP3-T133A transgenic mice were immunized with PBS or collagen as indicated for the secondary immunization. On day 52 after primary immunization, the paws were photographed. (**b**,**c**) At indicated times after the first immunization, the clinical scores (**b**) and paw thickness (**c**) were evaluated and recorded. The data were presented as mean±s.d. of five mice per group. ns, not significant; ***P<0.001 (analysis of variance (ANOVA), as compared with wild-type group; on day 50, 60 and 70, respectively). (**d**) On day 120 after primary immunization, whole paw joints were examined by H&E staining. Arrow heads denote the sites of bone and cartilage erosions. B, bone; C, cartilage; P, pannus; S, synovium. (**e**) Histomorphometric quantification of bone and cartilage erosions in joint tissues, corresponding to stainings in **d**. The data were presented as mean±s.d. of five high fields (ANOVA, as compared with wild-type group). (**f**) On day 52 after primary immunization, IL-6 levels in serum were examined by ELISA. The data were presented as mean±s.e. of five mice per group (ANOVA, as compared with wild-type group). All experiments were repeated three times.

We immunized RasGRP3 or RasGRP3-T133A transgenic mice with type II collagen of chicken on days 0 and 21, and found that the severity of arthritis in RasGRP3 transgenic mice was significantly regressed as compared with wild-type mice (day 52 after the primary immunization; Fig. 9a). Clinical scores and paw thickness data also indicated that RasGRP3 transgenic mice had regressed development of CIA (Fig. 9b,c). In the RasGRP3-T133A group, the development of CIA was enhanced, as demonstrated by persistent severe clinical features (Fig. 9a–c). RasGRP3 transgenic mice showed less pannus erosion of the bone and cartilage than wild-type mice (day 120), and clearer joint cavity and less damage of synovial tissue (Fig. 9d,e). In RasGRP3-T133A transgenic mice, the pannus erosions of the bone and cartilage, as well as the alterations of joint cavity and synovial tissue, were more severe than in the wild type (Fig. 9d,e).

Moreover, we found that the levels of IL-6 in the serum of RasGRP3 transgenic mice were significantly lower and of RasGRP3-T133A transgenic mice were significantly higher than of wild-type mice (day 52 after the primary immunization; Fig. 9f). These data indicate that RasGRP3 limits tissue damage during arthritis development.

### RasGRP3 knockout in RAW264.7 cells promotes IL-6 production

**Figure 10:**
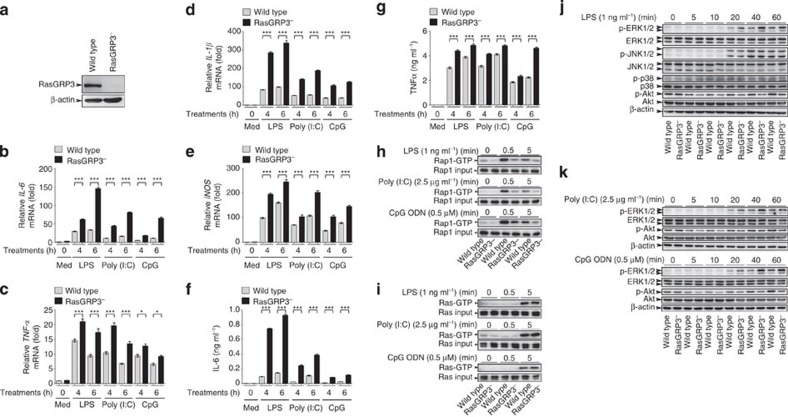
RasGRP3 deficiency promotes IL-6 production, impairs Rap1 activation and enhances ERK1/2 activation. (**a**) RAW264.7 cells cotransfected with the guide RNA-encoding plasmid and Cas9-encoding plasmid were sorted and selected for single clones. The depletion of RasGRP3 was examined by western blotting. (**b**–**g**) Wild-type RAW264.7 cells or RasGRP3-deficient RAW264.7 cells (RasGRP3^−^) were treated with or without (Med) 1 ng ml^−1^ LPS, 2.5 μg ml^−1^ Poly (I:C) or 0.5 μM CpG ODN as indicated. Next, *IL-6* (**b**), *TNF*α (**c**), *IL-1*β (**d**) and *iNOS* (**e**) mRNA levels were determined by Q-PCR, and IL-6 (**f**) or TNFα (**g**) in the culture supernatants were measured by ELISA. Data were presented as mean±s.d. of triplicate samples. *P<0.05; **P<0.01; ***P<0.001 (ANOVA). Levels of Rap1-GTP (**h**) and Ras-GTP (**i**) after LPS, Poly (I:C) or CpG ODN treatments as indicated. Extracts loaded with GDP or GTPγS were used as negative or positive controls for Rap1-GTP and Ras-GTP as recommended. (**j**,**k**) Wild-type or RasGRP3^−^ RAW264.7 cells were treated with LPS, Poly (I:C) or CpG ODN for different times, and the activation of indicated signalling molecules in whole-cell lysates were evaluated by western blotting. All experiments were repeated three times.

We have demonstrated the negative role of RasGRP3 in TLR-induced inflammatory response by using overexpression, knockdown or transgenic overexpression of RasGRP3. To confirm these findings, we depleted RasGRP3 in RAW264.7 cells using the genome-editing technique, the clustered regulatory interspersed short palindromic repeat (CRISPR)/CRISPR-associated protein (Cas) system. Four guiding RNA (gRNA) sequences were tested, and only one gRNA successfully depleted RasGRP3, which was confirmed by DNA sequencing and western blotting (Fig. 10a).

In RasGRP3-deficient RAW264.7 cells (RasGRP3^−^), the production of proinflammatory mediators such as IL-6, TNFα, IL-1β and iNOS was significantly increased after LPS, Poly (I:C) or CpG ODN treatments (Fig. 10b–g). For mechanistic studies, we examined the GTP-bound status of Rap1 and Ras, as well as the activation of MAPK and Akt. We found that RasGRP3^−^ RAW264.7 cells showed decreased levels of Rap1-GTP, accelerated dephosphorylation of Akt and enhanced ERK1/2 activation (Fig. 10h–k). However, the activation of Ras and the JNK1/2 and p38 signalling pathways were not affected by RasGRP3 depletion (Fig. 10i–k). These data suggest that RasGRP3 is a negative regulator of TLR signalling pathway and limits production of proinflammatory cytokines by activating Rap1 but promoting ERK1/2 activation in macrophages.

To exclude the off-target effects of RasGRP3 depletion, we overexpressed wild-type RasGRP3 and RasGRP3-T133A in RasGRP3^−^ cells (Supplementary Fig. 11). We found that RasGRP3, but not dominant-negative RasGRP3-T133A, could rescue the effects of RasGRP3 depletion on the production of proinflammatory cytokines after low-dose TLR3/4/9 agonist treatments (Supplementary Fig. 11a–f).

## Discussion

Proper response of host immune cells to TLR agonists is crucial for maintaining immunity, and is required for efficient clearance of pathogens and protection of host from overwhelming inflammation and autoimmunity^1^,^3^. Negative regulators of TLR signalling have not been completely elucidated^3^. Our study demonstrates that RasGRP3, a regulator of both Ras and Rap1, negatively regulates the production of proinflammatory cytokines (especially IL-6) by activating Rap1 and inhibiting ERK1/2 activation in response to low levels of TLR agonists, which may serve as an early regulatory machinery to limit inflammatory response of macrophages to feeble infection. The negative effects of RasGRP3 in regulating TLR responses on low levels of TLR ligands may represent innate ‘braking’ machinery for keeping macrophages from overactivation in case the infection is not fatally dangerous.

RasGRP family members are highly expressed in immune cells^14^,^15^. RasGRP1/3 are predominant in T and B cells, and play a critical role in the development of T and B cells, respectively^18^,^19^,^22^,^23^. RasGRP2 is mainly detected in platelets and can promote coagulation^21^. RasGRP4 is initially reported in mast cells and may be involved in regulation of allergy and asthma^24^,^25^. Theoretically, all the RasGRP members may be activated by TLR ligation, as it has been suggested that TLR signalling could induce production of DAG and increase of intracellular calcium^1^,^3^. We found that RasGRP3, but not RasGRP1, 2 or 4, inhibited TLR-triggered proinflammatory cytokine production in macrophages, which may be explicated by the abundant expression of RasGRP3 in macrophages. Moreover, DGKs, which converts DAG to phosphatidic acid, are expressed in a biased pattern in macrophages. Macrophages preferentially express DGK-α and DGK-ζ (negative regulator of RasGRP1), while DGK-ı (negative regulator of RasGRP3) is rarely detected in macrophages^34^,^35^,^36^, which may permit the activation of RasGRP3 by TLR-induced production of DAG.

Roles of Ras-related small GTPases in TLR signalling have not been clearly defined. Limited data have been obtained for Ras, Rac and Rab small GTPases in TLR responses, whereas the roles of Rap1 in TLR innate response have not been elucidated^5^,^6^,^7^,^8^,^9^,^10^,^11^. One report using splenocytes or CD3^+^ T cells showed that Rap1a deficiency increased the production of TNFα on ionomycin or PMA treatments^37^. Previous studies using transgenic mice or knockout mice have suggested that Rap1a is involved in the regulation of T-cell adhesion and immunological synapse formation^38^,^39^,^40^,^41^,^42^. In neutrophils and macrophages, Rap1 has also been implicated in cell adhesion and regulation of phagocytosis^43^,^44^,^45^,^46^,^47^. These studies clearly demonstrated an important role of Rap1 in integrin signalling, that is, ‘inside-out’ signalling of integrins. As compared with previous reports, our study has established the roles of Rap1 activation in outside-in TLR signalling: Rap1 activated by RasGRP3 negatively regulates TLR-triggered inflammatory cytokine production. Rap1 has once been considered as an antagonizing small GTPase for Ras^12^,^13^,^14^. It is possible that RasGRP3-Rap1-GTP antagonizes Ras-mediated activation of TLR signalling, leading to suppressed IL-6 production. However, our data do not support this proposal. We found that RasGRP3 could activate Rap1 but did not affect Ras-GTP levels in response to low levels of TLR agonists. Therefore, our study indicated that Rap1-GTP activated by RasGRP3 elicits negative regulation of TLR signalling through a Rap1-mediated negative signalling pathway. At least in our system (low levels of TLR agonists), RasGRP3, through selective activation of Rap1, does not regulate Ras signalling on TLR activation. The reasons why RasGRP3 selects Rap1 but not Ras in macrophages need further investigation. We propose here that the different sensitivity of Ras and Rap1 to low levels of active RasGRP3 may contribute to this selectivity of RasGRP3.

Macrophages are always at the first line of defense against pathogen infection. One key question regarding TLR signalling is that how macrophages decide to be or not to be activated by TLR ligation. In most of studies, high levels of TLR agonists are used in determining the response of macrophages to TLR activation. However, especially under physiological conditions, macrophages may continuously encounter endogenous or exogenous TLR ligands. Thus, macrophages need sufficient negative regulatory molecules to avoid frequent activation. Our study using the knockdown assays and transgenic mice/cells suggests that RasGRP3-mediated Rap1 activation may be such a ‘brake’ for macrophages. At higher and usual doses of TLR agonists, RasGRP3 failed to inhibit production of inflammatory cytokines, while only at low doses of TLR agonists, RasGRP3-mediated Rap1 activation elicited negative effects on IL-6 production. Therefore, RasGRP3 may set a threshold for macrophages to produce IL-6 by activating Rap1, thus avoiding frequent activation of macrophages under physiological conditions.

Typically, TLRs activate downstream signalling pathways, including MAPK, NF-κB and TBK1-IRF3, via the MyD88- and/or TRIF-dependent mechanisms^1^,^3^. In our study, we observed increased ERK1/2 activation in RasGRP3-silenced macrophages after TLR4, TLR3 and TLR9 ligations. In contrast, other MAPKs (JNK and p38), NF-κB and TBK1-IRF3 pathways were not significantly affected by RasGRP3. MEK/ERK inhibitor reversed the effects of RasGRP3 knockdown, indicating that the observed increase of IL-6 production may be due to the effects of RasGRP3 knockdown on ERK1/2 activation. Rap1 has been previously shown to activate MEK/ERK via BRAF^48^, which is in contrast to RasGRP3 knockdown-induced Rap1 inhibition but ERK1/2 activation, thus indicating that RasGRP3-Rap1 may regulate ERK1/2 indirectly and independently on Rap1-mediated BRAF/MEK/ERK activation. One interesting finding in our study is the negative correlation of Akt with ERK1/2 activation. After RasGRP3 knockdown, the dephosphorylation of Akt was accelerated and the rephosphorylation of Akt was inhibited, leading to lower overall level of Akt activation. Meanwhile, ERK1/2 activation was enhanced by RasGRP3 knockdown. Therefore, RasGRP3 knockdown-induced ERK1/2 activation may be due to the impaired Akt phosphorylation. Thus, we inferred here that RasGRP3 may negatively regulate the ERK1/2-mediated proinflammatory cytokine production via impairing the dephosphorylation but promoting the rephosphorylation of Akt, which may retain the Akt at higher active state, and, in turn, prevent ERK1/2 activation. Previously, PI3K/Akt signalling pathway has been suggested to negatively regulate TLR-induced production of inflammatory cytokines^31^. Thus, RasGRP3-Rap1-mediated increase in phospho-Akt levels may lead to suppressed production of IL-6. However, how the phospho-Akt prevents the activation of ERK1/2 has not been resolved by our study and will require further investigation.

Previously, RasGRPs have been implicated in autoimmune diseases such as systemic lupus erythematosus (SLE) and RA^18^,^25^,^49^,^50^,^51^,^52^,^53^,^54^. It has been reported that RasGRP1-null mice develop lymphoproliferative disorders that resemble that seen in patients with SLE^18^, supported by later findings that defective expression of functional RasGRP1 and abnormal overexpression of *RasGRP1* isoforms can be detected in SLE patients^49^,^50^. RasGRP4-null mice demonstrated less severe inflammation in experimental colitis and arthritis^25^, indicating an essential role of RasGRP4 in the development of inflammatory diseases. Similar to RasGRP1, aberrant splicing of RasGRP4 has been detected in SLE patients^51^. RasGRP3 is also associated with clinical features of SLE by using genome-wide association studies^52^,^53^,^54^. Our data demonstrate a negative role of RasGRP3 in regulating proinflammatory cytokine production by macrophages on low levels of TLR agonist stimuli. Our study suggests a potential protective role of RasGRP3 in inflammatory diseases. We demonstrate that RasGRP3 transgenic overexpression inhibits TLR agonists-induced IL-6 production, decreases the inflammation severity of DSS-induced colitis and relieves the clinical scores of CIA. Functionally, RasGRP3 may be more alike to RasGRP2, both of which can activate Rap1 (refs 20, 28). The contrast effects of RasGRP3 on experimental colitis and arthritis, as compared with RasGRP4 (ref. 25), may thus be attributed to differential selection of Rap1 or Ras as substrates. Whether RasGRP2 contributes to inflammatory diseases as colitis or arthritis, although expectable, may need investigations in the future. It should be noted that most of our data are obtained in transgenic mice model, which may need further confirmation in conditional knockout mice model (unavailable at present). The reconstitution experiments using bone marrow transplantation and transfer of transgenic macrophages in irradiated wild-type mice have greatly strengthened our study. Because of long observation duration of the CIA mice model, reconstitution experiments were not finished at present. However, based on our *in vitro* and *in vivo* studies, we conclude that RasGRP3 is a negative regulator of TLR-triggered inflammatory response and may protect host from inflammatory diseases, at least macrophage-dedicated inflammatory diseases. The G558R mutation of RasGRP3 detected in a colon cancer patient indicates that RasGRP3 may be associated with inflammation-related diseases, which may need validations in clinical samples derived from patients with SLE, RA or IBD.

In summary, our study has demonstrated that RasGRP3 can limit TLR-triggered proinflammatory cytokines (especially IL-6) production, possibly through the Rap1-Akt-ERK1/2 pathway. Under physiological conditions, macrophages are subjected to low amounts of pathogenic microorganisms, whose concentrations are far below the usual doses of stimuli in studies of TLR signalling. RasGRP3 may play an important role in regulating TLR response of macrophages under physiological conditions, thus setting a threshold for macrophages to be activated and decreasing the probability of inflammatory diseases.

## Methods

### Mice and reagents

Wild-type C57BL/6 mice (6–8 weeks of age) were obtained from Jackson Laboratories (Bar Harbor, Maine). All the animal experiments were approved by the Medical Ethics Committee of the Zhejiang University School of Medicine and conducted according to the Declaration of Helsinki Principles. Poly (I:C) and LPS (0111:B4) were purchased from Sigma (St. Louis, MO). C-class CpG ODN (ODN 2395) was obtained from Invivogen (San Diego, California). Antibodies specific to Flag-tag (ab125243), phospho-RasGRP3 (Thr133) (ab124843), RasGRP1 (ab37927) and RasGRP2 (ab137608) were from Abcam Inc. (Cambridge, MA). Ab for RasGRP4 (sc-292930) was obtained from Santa Cruz Biotechnology (Dallas, TX). Ab specific for RasGRP3 (3334) and Abs against total and phosphorylated forms of ERK1/2 (Thr202/Tyr204) (4695, 9101), JNK1/2 (Thr183/Tyr185) (4668, 9252), p38 (Thr180/Tyr182) (9212, 9215), IKKα/β (Ser176/180) (2697, 8943), Akt (Ser473) (4060, 9272), Akt (Thr307) (9275) and IκBα (Ser32/36) (4814, 9246) were from Cell Signaling Technology (Beverly, MA). Ab for β-actin (A1987) was from Sigma.

### Construction of expression plasmids

The recombinant vectors encoding mouse RasGRP3 (GenBank number NM_001166493) and human RasGRP3 (GenBank number NM_001139488), and vectors encoding Flag-tagged RasGRP3 were constructed by PCR-based amplification, and then subcloned into the pcDNA3.1 eukaryotic expression vector (Invitrogen, San Diego, CA)^9^,^55^. Mutant forms of vectors, namely RasGRP3-T133A (mutation of Thr133 into Ala), RasGRP3-G558R (mutation of Gly558 into Arg) and H-RasG12V (mutation of Gly12 into Val), were prepared by using Mutanbest Kit according to manufacturer’s instructions (TaKaRa, Shiga, Japan). All the clones were confirmed by DNA sequencing. Primer sequences used in cloning are listed in Supplementary Table 1.

### Cell preparation, cell culture and transfection

RAW264.7 macrophages (ATCC, Manassas, VA) were cultured in Roswell Park Memorial Institute (RPMI)-1640 medium containing 10% FCS (Gibco of Life Technologies, Grand Island, NY). For preparation of peritoneal macrophages, 1 ml PBS containing 3% sodium thioglycollate (Sigma) was intraperitoneally injected into abdominal cavity of each C57BL/6 mouse. Five days later, cells in the abdominal cavity were collected from 5 ml PBS injected intraperitoneally and then by centrifugation, and were allowed to adhere to plastic 100-mm culture dish (Corning Costar, Tewksbury, MA) for 12 h in complete RPMI-1640 medium. After removing floating cells by adding and aspirating 0.5 ml RPMI-1640 medium twice, peritoneal macrophages adhered to the dish were collected. Bone marrow-derived macrophages were obtained by culturing thigh bone-derived bone marrow cells in complete RPMI-1640 medium containing 20 ng ml^−1^ recombinant macrophage colony-stimulating factor (R&D Systems, Minneapolis, MN) for 5 days^9^,^55^. For the culture of human MDM cells derived from healthy peripheral blood, CD14^+^ cells were isolated from mononuclear cells by using magnetic beads (Miltenyi Biotec Technology & Trading, Bergisch Gladbach, Germany) and cultured with 20 ng ml^−1^ recombinant macrophage colony-stimulating factor (R&D Systems) for 5 days^56^. For transient transfection of plasmids in RAW264.7 cells, the jetPEI reagents were used according to the manufacturer’s instructions (Polyplus-Transfection Company, Illkirch, France).

### Quantitative PCR

Total cellular RNA was extracted using Trizol reagent (Invitrogen) and complementary DNA was synthesized by using the AMV Reverse Transcriptase kit (Promega, Madison, WI). Specific primers used for quantitative PCR (Q-PCR) assays are listed in Supplementary Table 2. Q-PCR was performed on a MJR Chromo 4 Continuous Fluorescence detector (Bio-Rad, Hercules, CA) according to the manufacturer’s protocol. The levels of indicated molecules were determined by evaluating the threshold cycle (Ct) of target gene after normalization against the Ct value of β*-*actin and calculated by using the formula 2^−(Ct of target gene−Ct of β*-*actin)^ (refs 9, 55).

### RNA interference

We have synthesized and selected four small interfering RNA (siRNA) duplexes (Shanghai GenePharma Co., Shanghai, China) for the knockdown of each target, including RasGRP1-4, Rap1a and Rap1b. For transient knockdown, 21-nucleotide sequences of siRNAs were synthesized (Supplementary Table 3). siRNA duplexes were transfected into macrophages using INTERFERin-HTS according to the standard protocol (Polyplus-Transfection Company). Forty-eight hours later, the efficiency of RNA interference was determined by Q-PCR and/or western blot assays.

### Measurement of cytokines

ELISA kits for mouse and human IL-6 and TNFα were obtained from R&D Systems. Cytokine concentrations in culture supernatants or serum were measured by ELISA as recommended^9^,^55^. Briefly, 50 μl of diluted culture supernatants (tenfold) or serum (fourfold) were added to 50 μl of assay diluent in each well, mixed gently and incubated for 2 h at room temperature. After adding 100 μl per well of horseradish peroxidase-conjugated anti-IL-6 or anti-TNFα antibody and incubating the plate for 2 h at room temperature, 100 μl per well of substrate was added to the extensively washed plate. Finally, the optical intensity of each sample was determined by subtracting readings at 540 nm from the readings at 450 nm. Concentration of cytokines was calculated by using the formula established by the standard curve of standard samples.

### Western blotting

Total cell lysates were prepared by using cell lysis buffer (Cell Signaling Technology) containing phosphatase inhibitor cocktail (Sigma)^9^,^55^ and protein concentration determined by the BCA protein assay (Pierce, Rockford, IL). Cell extracts containing equal amounts of proteins were subjected to SDS–PAGE, transferred onto nitrocellulose membrane and blotted as per the standard protocol^9^,^55^. The primary antibodies and horseradish peroxidase-conjugated secondary antibodies were used at a final concentration of 100 ng ml^−1^ diluted in a buffer containing 50 mM Tris.HCl (pH 7.4), 150 mM NaCl and 0.05% Tween 20. The bands were revealed using Supersignal West Femto Maximum Sensitivity substrate (Pierce) and were imaged and analysed by using Syngene Bio Imaging Systems (Frederick, MD). Full-sized scans of western blottings are provided in Supplementary Fig. 12.

### Determination of GTP-bound Ras and Rap1 levels

The examinations of Ras-GTP and Rap1-GTP levels were performed using the Ras Activation Assay Kit and the Rap1 Activation Assay Kit, respectively, as recommended by the manufacturer (Merck Millipore, Billerica, MA). The levels of GTP-bound Ras and Rap1 were evaluated by measuring the precipitated proteins by Raf-1 RBD agarose and Ral GDS RBD agarose, respectively. Briefly, extracts of macrophages were incubated with 10–30 μl of Raf-1 RBD agarose or Ral GDS RBD agarose for 45 min at 4 °C. After being washed three times in lysis buffer, the agaroses were resuspended in 40 μl of 2 × Laemmli buffer containing 50 mM dithiothreitol. After boiling the samples for 5 min, western blot assays were performed to examine Ras or Rap1 as suggested. Extracts loaded with GTPγS or GDP were used as positive and negative control, respectively.

### CRISPR-Cas9-mediated depletion of RasGRP3

For the depletion of RasGRP3 in RAW264.7 cells, pc3-U6-guide RNA-CMV-RED (encoding gRNA and red fluorescent protein) and Cas9-IRES-EGFP (encoding Cas9 and green fluorescent protein) plasmids (kind gifts from Shanghai Biomodel Organism Science & Technology Development Co., Shanghai, China) were cotransfected into RAW264.7 cells. Four target sequences for gRNA synthesis were tested: 5′-GAGGAAAAAAGTATCCAAAA-3′, 5′-GAATCGTGTTACTGATGCACCGA-3′, 5′-CTGAACTGGCAGGAAAACTC-3′ and 5′-GAACGACAGTTACTTGCCCAGAA-3′. After ligation of synthesized sequences into the pc3-U6-guide RNA-EGFP and cotransfection of the two plasmids into RAW264.7 cells, cells with both red and green fluorescence were then sorted by using Gallios Flow Cytometer (Beckman Coulter, Brea, CA). Sorted cells were cultured for 3–5 days and clones propagated from single cell were picked out. The depletion of RasGRP3 was confirmed by both western blotting and DNA sequencing. Only the second target sequence could efficiently deplete RasGRP3 expression. The selected clones demonstrating unchanged RasGRP3 expression were used as wild-type clones, while the clones deficient for RasGRP3 were used as RasGRP3^−^ clones.

### Generation of RasGRP3 transgenic mice

The fertilized eggs were microinjected with the linearized vectors encoding Flag-tagged full-length RasGRP3 or RasGRP3-T133A under the control of cytomegalovirus promoter and were transplanted into the oviduct of pseudo-pregnant C57BL/6 mice (Shanghai Biomodel Organism Science & Technology Development Co.)^55^. After the birth of transgenics, the founder mice were identified by PCR assays of genomic DNA derived from the tail of transgenic mice and hybridized with wild-type C57BL/6 mice to produce animals used for all the experiments.

### *In vivo* modulation of TLR response

Indicated amounts of LPS, Poly (I:C) or CpG ODN in 200 μl of LPS were injected intraperitoneally. Serum concentrations of IL-6 and TNFα were determined by ELISA.

### DSS-induced colitis and colitis histology

Indicated 8- to 12-week-old C57BL/6 mice received water containing 2.5% DSS for 7 days and normal water later^57^. On indicated days, body weight of each mouse was measured. On day 10 after DSS treatments, sera were collected for ELISA assays of IL-6 and TNFα, and the colons were subjected to H&E analysis for inflammation severity. Colons were rinsed with cold PBS to remove fecal material, and tissue sections were fixed in 10% buffered formalin and embedded in paraffin. Five-micrometre-thick sections were stained with H&E. Colitis scores (0–4) were determined by a staff pathologist who did not know the experimental protocol. The criteria for evaluating colitis scores were as following^58^: score 0, no change from normal tissue; score 1, one or a few multifocal mononuclear cell infiltrates in the lamina propria accompanied by minimal epithelial hyperplasia and slight-to-no depletion of mucus from goblet cells; score 2, lesions tended to involve more of the intestine than grade 1 lesions, or were more frequent; score 3, lesions involved a large area of the mucosa, the submucosa but rarely transmural, or were more frequent than grade 2 lesions; score 4, lesions usually involved most of the intestinal section and were more severe than grade 3 lesions (sometimes transmural), epithelial hyperplasia marked with crowding of epithelial cells in elongated glands, loss of mucin-containing cells or presence of crypt abscesses and ulcers. Twenty separate microscopic fields were evaluated for each mouse.

### Immunization protocols and manipulation of CIA

To establish the model of CIA in RasGRP3 or RasGRP3-T133A transgenic mice and in wild-type mice, all mice (6 weeks of age) were immunized intradermally at the base of the tail with 100 μg of chicken type II collagen in complete Freunds’ adjuvant (Sigma)^59^. Twenty-one days after the primary immunization, the mice were boosted with a secondary immunization with same amount of type II collagen emulsified in incomplete Freunds’ adjuvant (Sigma) intradermally in the tail but proximal to the primary injection site. Next, the clinical scores for each paw were evaluated and scored individually on a scale of 0–4, two to three times per week^59^. On day 120, whole paw joints were fixed in 4% phosphate-buffered paraformaldehyde, decalcified in EDTA and then embedded in paraffin. Specimens were longitudinally cut into 4-μm sections for H&E staining (Sigma-Aldrich). The histological alterations were then examined under a microscope.

### Irradiation and transgenic macrophage transfer

Wild-type C57BL/6 mice were lethally irradiated with two doses of 500 Rads each mouse. Donor mice (wild-type, RasGRP3 or RasGRP3-T133A transgenic mice) were killed by CO_2_ inhalation and the bones (forelimbs, hindlimbs and hips) were flushed with sterile PBS to obtain bone marrow stem cells. Cells were injected intraperitoneally (2 × 10^6^ total cells per mouse) within 6–8 h after irradiation. On day 8, 2 × 10^6^ peritoneal macrophages derived from wild-type, RasGRP3 or RasGRP3-T133A transgenic mice were intraperitoneally injected into the irradiated mice. On day 10, the mice were subjected to TLR agonists administration or DSS treatments *in vivo*.

### Statistical analysis

All the experiments were independently repeated at least three times. Results are given as mean±s.e. or mean±s.d. Comparisons between two groups were done using Student’s *t*-test or Mann–Whitney *U*-test. Multiple comparisons were done with one-way analysis of variance followed by Fisher’s least significant difference analysis. Statistical significance was determined as *P*<0.05.

## Author contributions

T.C., X.C. and J.W. designed the experiments and supervised the project; X.C. and T.C. wrote the manuscript; S.T., T.C. and Z.Y. prepared and cultured the cells; S.T. and T.C. performed Q-PCR and western blot assays; M.Y. and X.Z. established the transgenic mice and the mouse disease model; B.X. and N.L. constructed and validated the vectors.

## Additional information

**How to cite this article:** Tang, S. *et al.* RasGRP3 limits Toll-like receptor-triggered inflammatory response in macrophages by activating Rap1 small GTPase. *Nat. Commun.* 5:4657 doi: 10.1038/ncomms5657 (2014).

## Supplementary Material

Supplementary Information Supplementary Figures 1-12 and Supplementary Tables 1-3
