# Supplementary material for: RasGRP3 limits Toll-like receptor-triggered inflammatory response in macrophages by activating Rap1 small GTPase
Source: Nat Commun. 2014 Aug 14;5:4657. doi: 10.1038/ncomms5657 (PMC4143924; doi:10.1038/ncomms5657)
Supplement: Supplementary Information — Supplementary Figures 1-12 and Supplementary Tables 1-3 [file ncomms5657-s1.pdf]

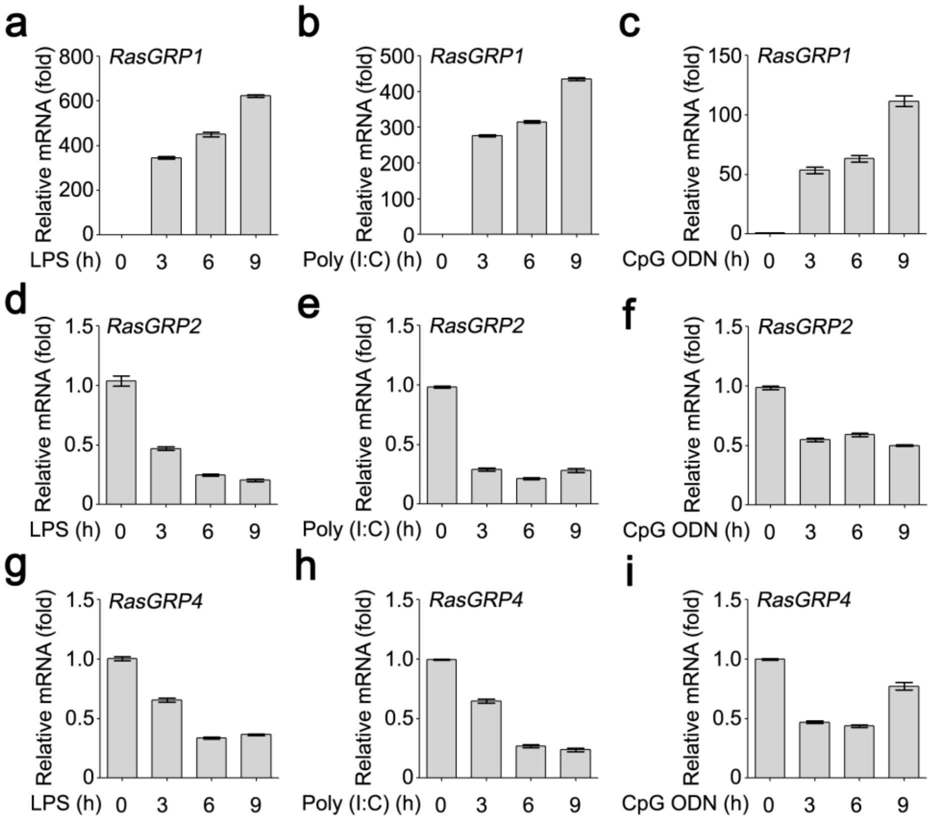

Supplementary Figure 1. RasGRP1, RasGRP2 and RasGRP4 expression levels in mouse macrophages. Peritoneal macrophages derived from C57BL/6 wild type mice were treated with or without 100 ng/ml LPS (**a**, **d**, **g**), 10 μg/ml Poly (I:C) (**b**, **e**, **h**) or 5 μM CpG ODN (**c**, **f**, **i**) as indicated. The mRNA levels of *RasGRP1* (**a-c**), *RasGRP2* (**d-f**) and *RasGRP4* (**g-i**) were examined by Q-PCR. Data are presented as mean±s.d. of triplicate samples and are representative of three independent experiments.

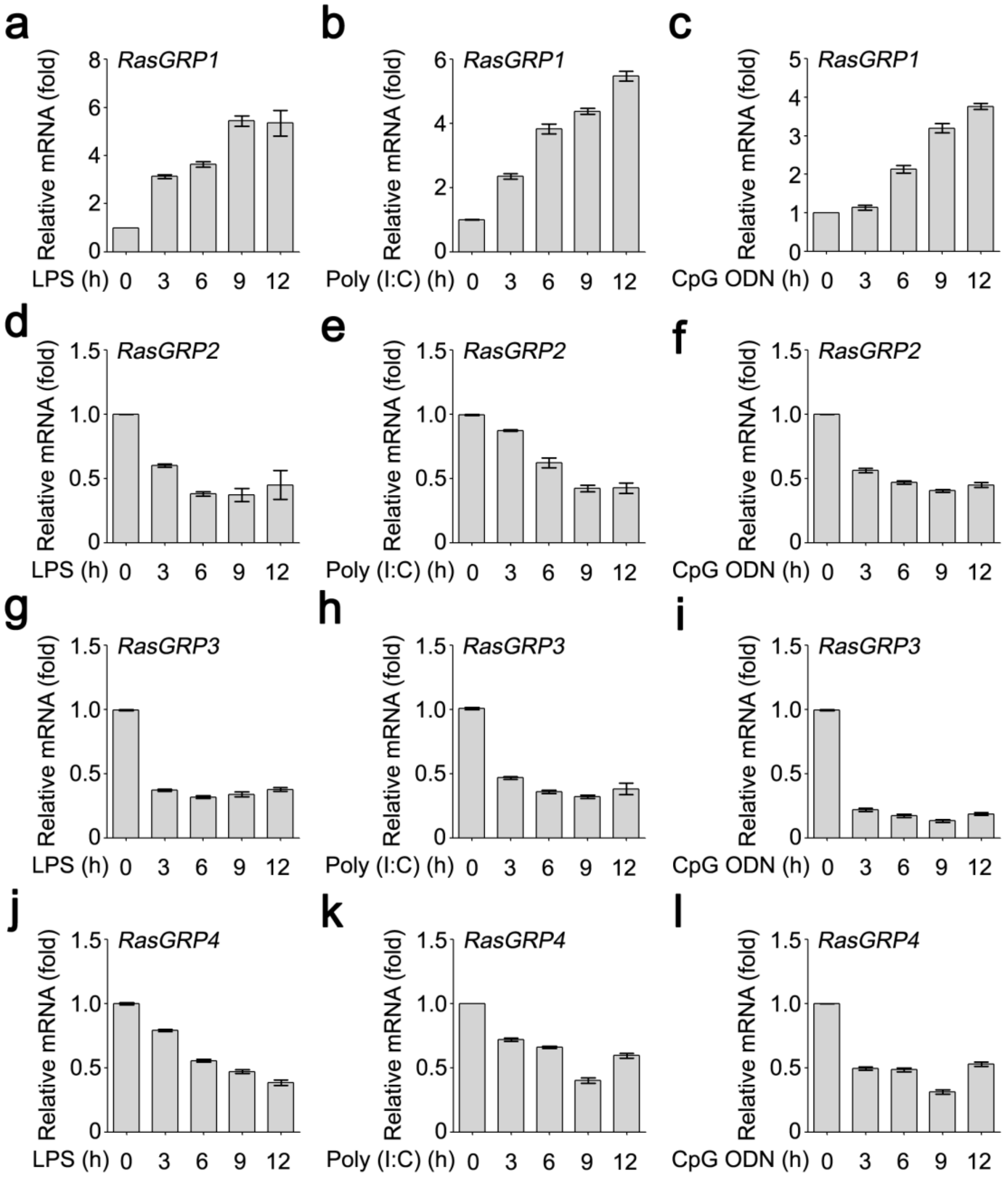

Supplementary Figure 2. RasGRP1-4 expression levels in PMA-differentiated THP-1 cells. THP-1 cells differentiated with 0.5 ng/ml PMA for 12h were treated with or without 100 ng/ml LPS (**a**, **d**, **g**, **j**), 10 μg/ml Poly (I:C) (**b**, **e**, **h**, **k**) or 5 μM CpG ODN (**c**, **f**, **i**, **l**) as indicated. The mRNA levels of *RasGRP1* (**a-c**), *RasGRP2* (**d-f**), RasGRP3 (**g-i**) and *RasGRP4* (**j-l**) were examined by Q-PCR. Data are presented as mean±s.d. of triplicate samples and are representative of three independent experiments.

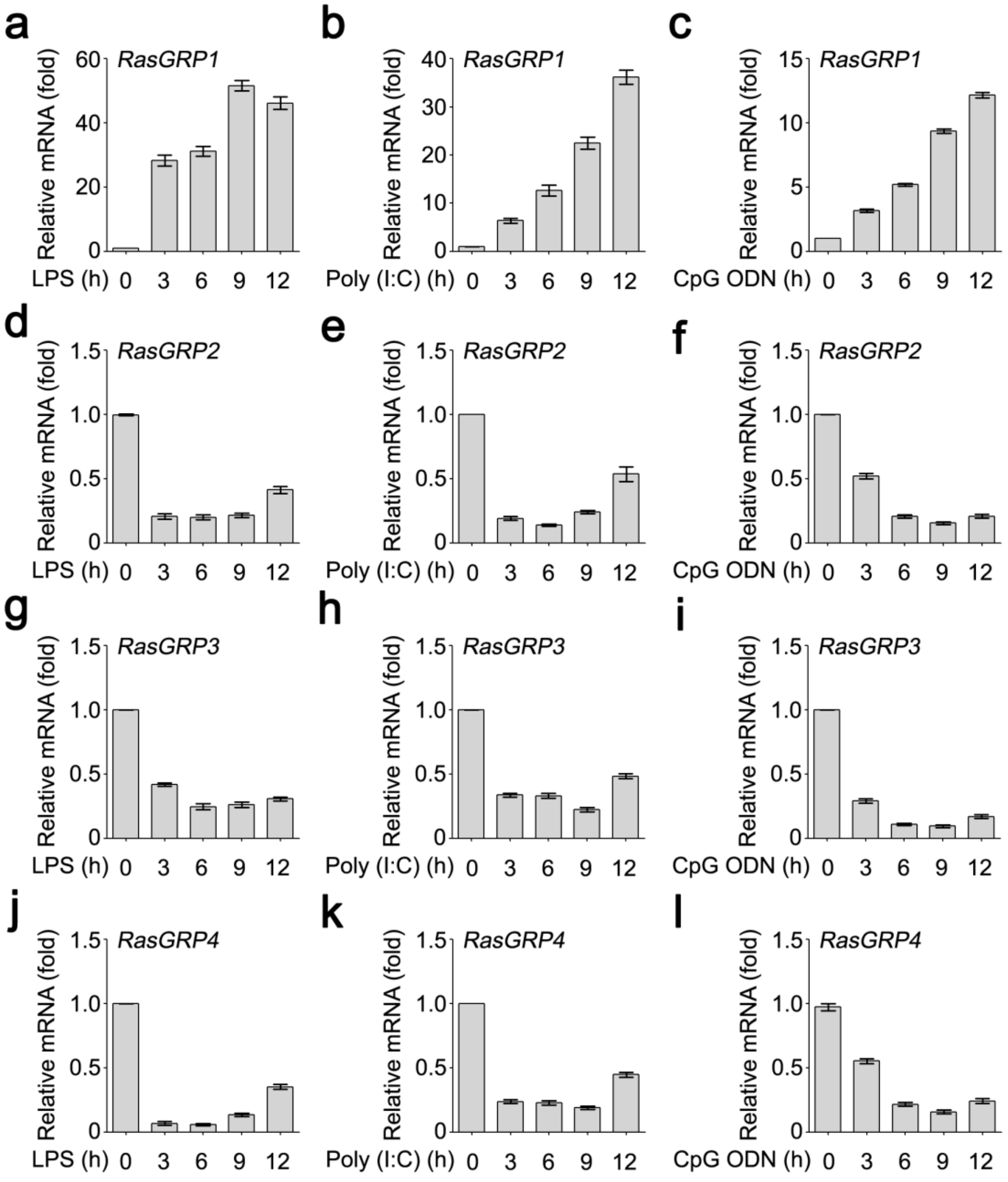

Supplementary Figure 3. RasGRP1-4 expression levels in human monocyte-derived macrophages. Human monocyte-derived macrophages were treated with or without 100 ng/ml LPS (**a**, **d**, **g**, **j**), 10 μg/ml Poly (I:C) (**b**, **e**, **h**, **k**) or 5 μM CpG ODN (**c**, **f**, **i**, **l**) as indicated. The mRNA levels of *RasGRP1* (**a-c**), *RasGRP2* (**d-f**), RasGRP3 (**g-i**) and *RasGRP4* (**j-l**) were examined by Q-PCR. Data are presented as mean±s.d. of triplicate samples and are representative of three independent experiments.

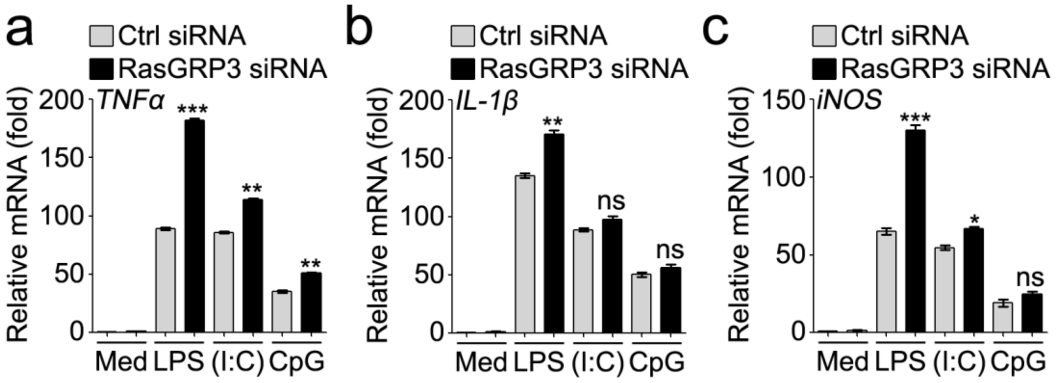

Supplementary Figure 4. Knockdown of RasGRP3 promotes the expression of *TNFα* and *iNOS* in macrophages. Peritoneal macrophages were transiently transfected with control (Ctrl) or RasGRP3-specific siRNAs for 48h. Cells were treated with 1 ng/ml LPS, 2.5 μg/ml Poly (I:C) or 0.5 μM CpG ODN for 2h (for *TNFα* mRNA) or 6h. Then mRNA levels of *TNFα* (**a**), *IL-1β* (**b**) and *iNOS* (**c**) were determined by Q-PCR. Data are presented as mean±s.d. of triplicate samples and are representative of three independent experiments. ns, not significant; *, $P < 0.05$; **, $P < 0.01$; ***, $P < 0.001$ (ANOVA).

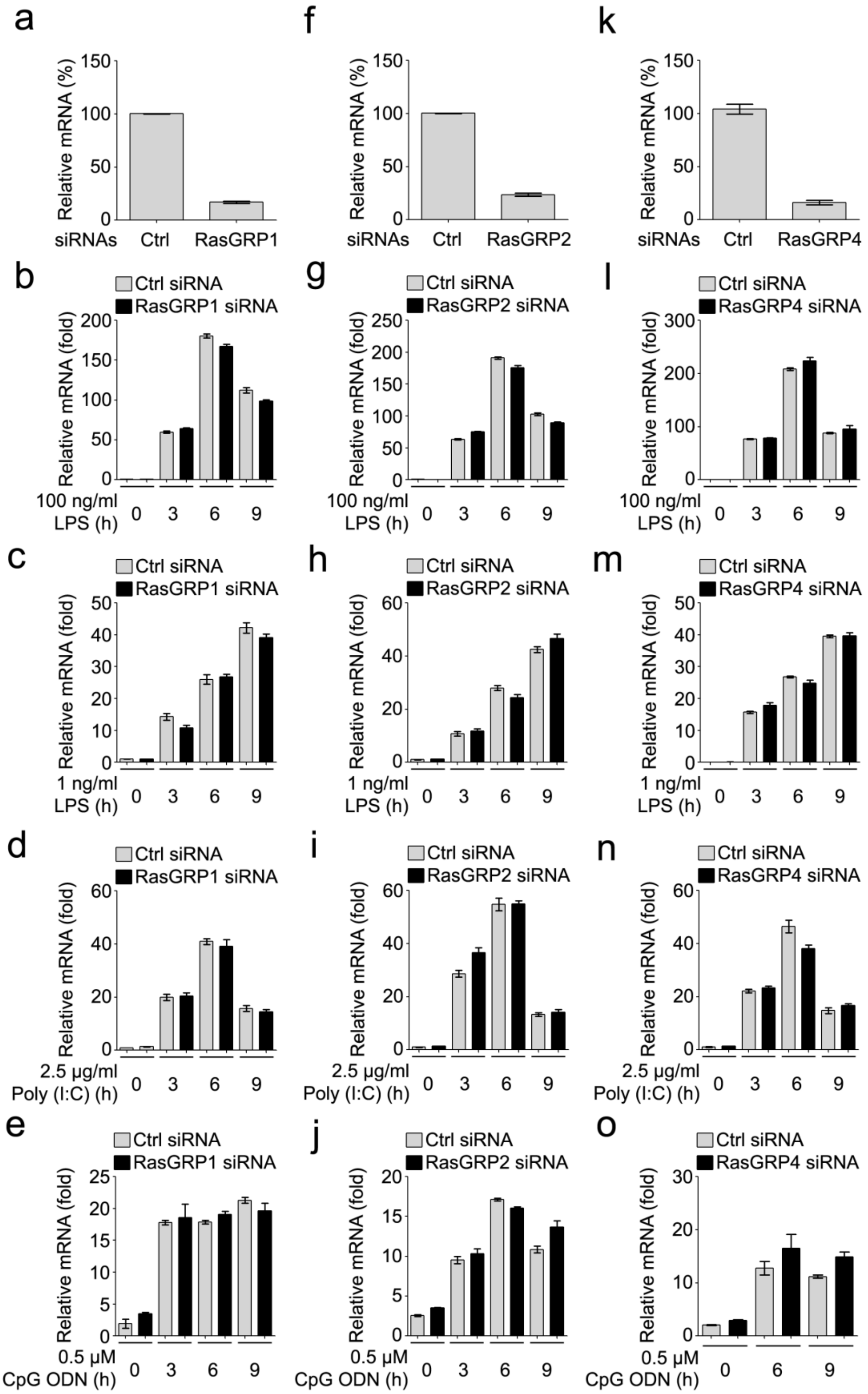

Supplementary Figure 5. Knockdown of RasGRP1, RasGRP2 or RasGRP4 does not affect IL-6 production in mouse macrophages. (**a-e**) Peritoneal macrophages

were transiently transfected with control (Ctrl) or siRNAs for RasGRP1 for 48h. Efficiencies of siRNAs were determined by Q-PCR (**a**). Cells were treated with 100 ng/ml (**b**), 1 ng/ml LPS (**c**), 2.5 μg/ml Poly (I:C) (**d**) or 0.5 μM CpG ODN (**e**) as indicated. Then mRNA levels of *IL-6* were determined by Q-PCR (**b-e**). (**f-j**) Peritoneal macrophages were transiently transfected with control (Ctrl) or siRNAs for RasGRP2 for 48h. Efficiencies of siRNAs were determined by Q-PCR (**f**). Cells were treated and then examined for *IL-6* mRNA as in (**b-e**). (**k-o**) Peritoneal macrophages were transiently transfected with control (Ctrl) or siRNAs for RasGRP4 for 48h. Efficiencies of siRNAs were determined by Q-PCR (**k**). Cells were treated and then examined for *IL-6* mRNA as in (**b-e**). Data are presented as mean±s.d. of triplicate samples and are representative of three independent experiments.

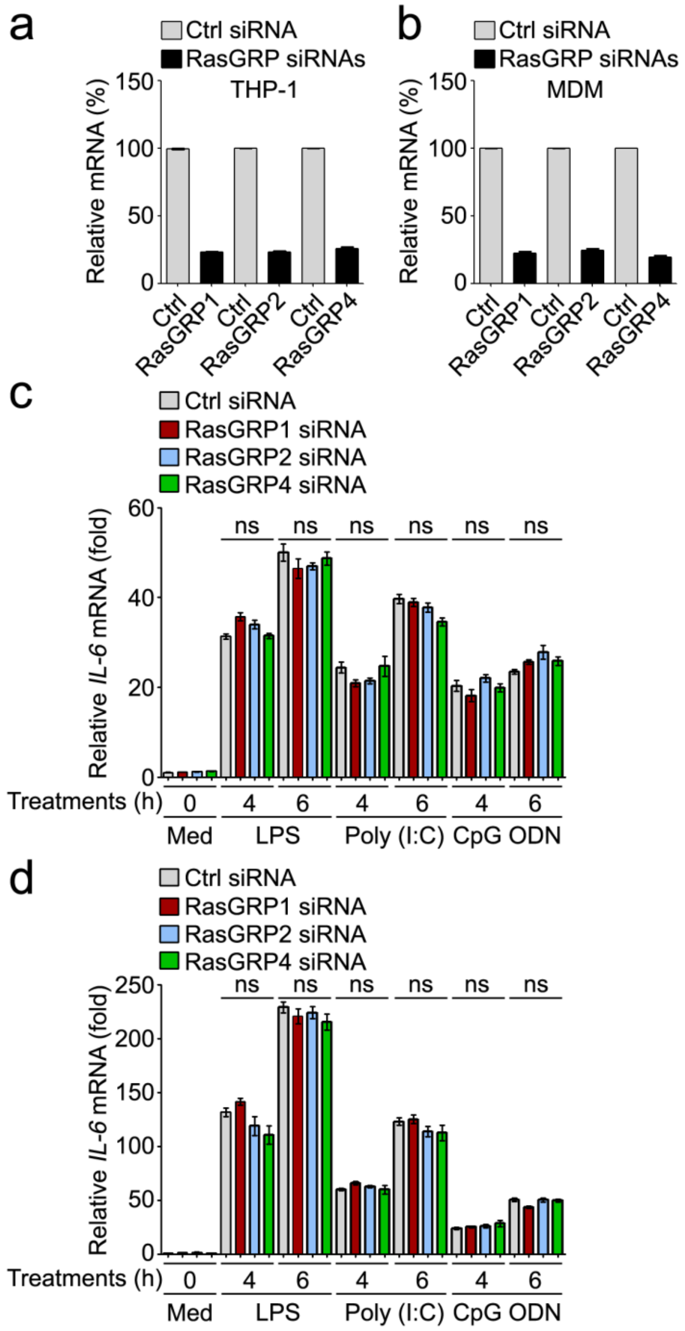

Supplementary Figure 6. Knockdown of RasGRP1, RasGRP2 or RasGRP4 does not affect IL-6 production in human macrophages. (**a**, **b**) THP-1 cells differentiated with 0.5 ng/ml PMA for 12h (**a**) or human monocyte-derived macrophages (**b**) were transiently transfected with control (Ctrl) or siRNAs for *RasGRP1*, *RasGRP2* or *RasGRP4* for 48h. Efficiencies of siRNAs were determined by Q-PCR. (**c**, **d**) THP-1 cells (**c**) as in (**a**) and MDM (**d**) as in (**b**) were treated with or without (Med) 1 ng/ml LPS, 2.5 μg/ml Poly (I:C) or 0.5 μM CpG ODN as indicated. Then mRNA levels of *IL-6* were determined by Q-PCR. Data are presented as mean±s.d. of triplicate samples. ns, not significant (ANOVA).

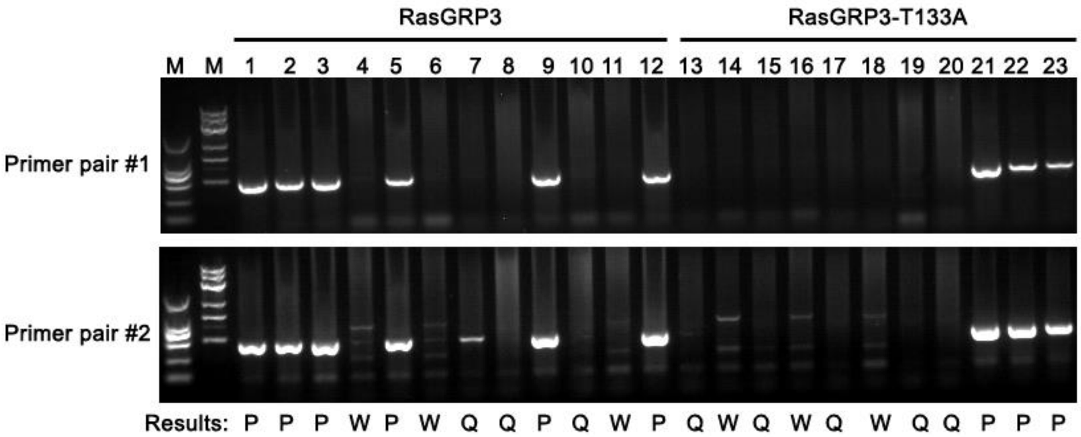

Supplementary Figure 7. Characterization of RasGRP3 transgenic mice. Genomic DNA derived from tails of RasGRP3 or RasGRP3-T133A transgenic mice were examined by PCR. The PCR results were evaluated as indicated in the bottom ("P" for positive, "W" for wild type, and "Q" for suspicions of DNA degradation or low DNA concentration that need a second validation using another DNA sample). Only the mice showing strong positive results by using both pairs of primers were regarded as transgenic mice and used in the study.

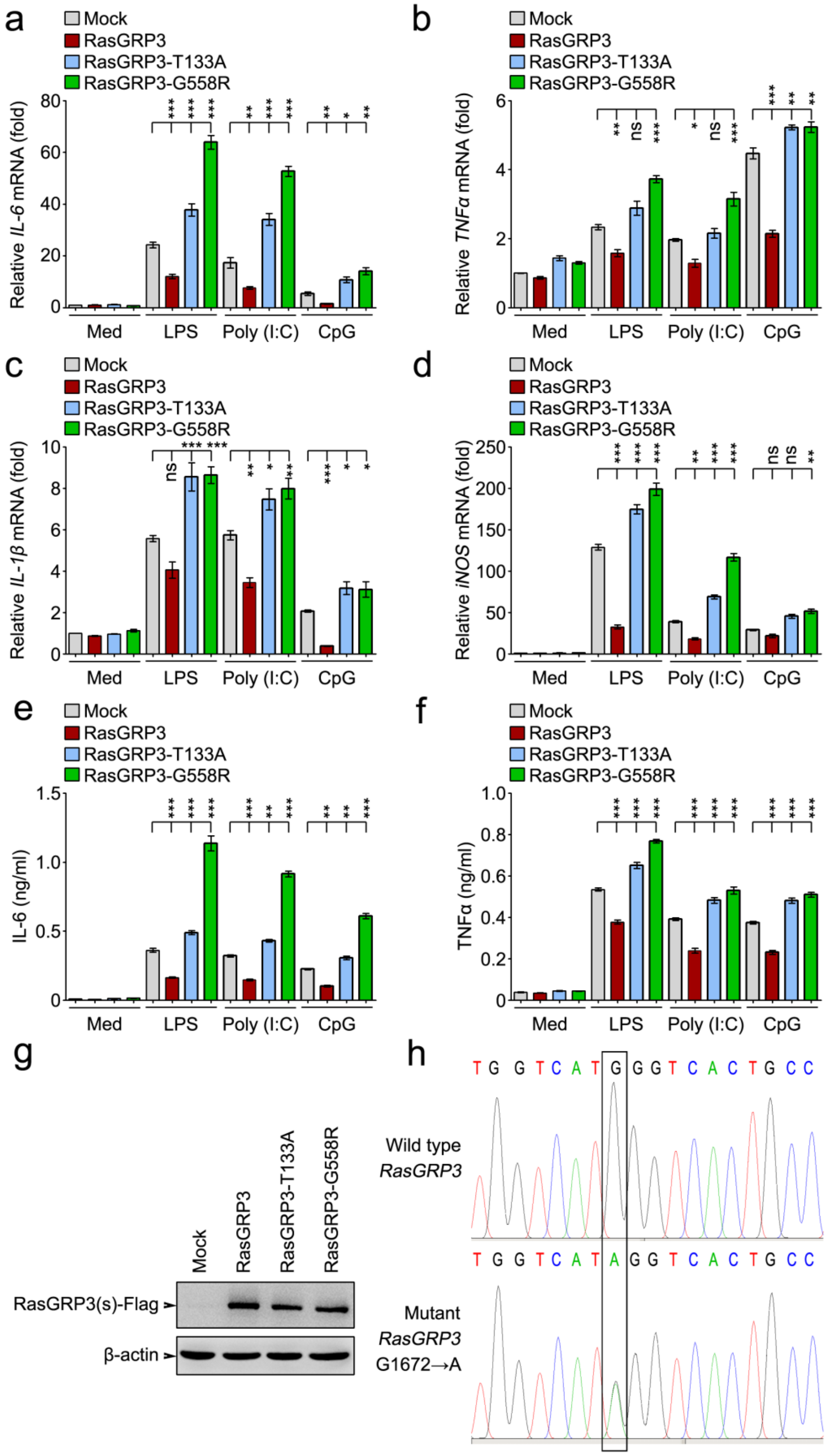

Supplementary Figure 8. RasGRP3 inhibits production of inflammatory

cytokines in THP-1 cells. (**a-f**) THP-1 cells differentiated with 0.5 ng/ml PMA for 12h were treated with or without (Med) 1 ng/ml LPS, 2.5 μg/ml Poly (I:C) or 0.5 μM CpG ODN as indicated. Then mRNA levels of indicated factors were determined by Q-PCR (**a-d**). IL-6 and TNFα in the culture supernatants were measured by ELISA (**e**, **f**). Data are presented as mean±s.d. of triplicate samples. ns, not significant; *, $P < 0.05$; **, $P < 0.01$; ***, $P < 0.001$ (ANOVA). (**g**) Western blot assays of RasGRP3 (mutant) overexpression. (**h**) DNA sequencing results of a RasGRP3 mutation (G1672 mutated to A, resulting in G558R mutation of RasGRP3) in a colon cancer patient. The mutated nucleic acid was boxed. Data are representative of three independent experiments.

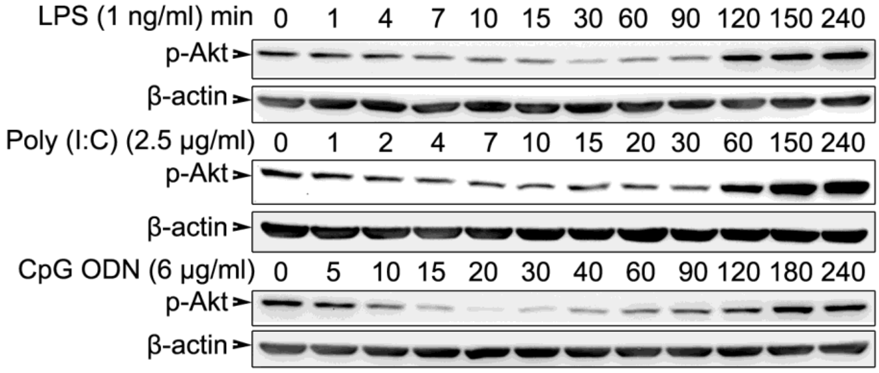

Supplementary Figure 9. The dephosphorylation and rephosphorylation of Akt after treatments with low levels of TLR agonists in macrophages. Peritoneal macrophages were treated as indicated. Phosphorylation of Akt (Ser473) was evaluated by Western blot. Data are representative of three independent experiments.

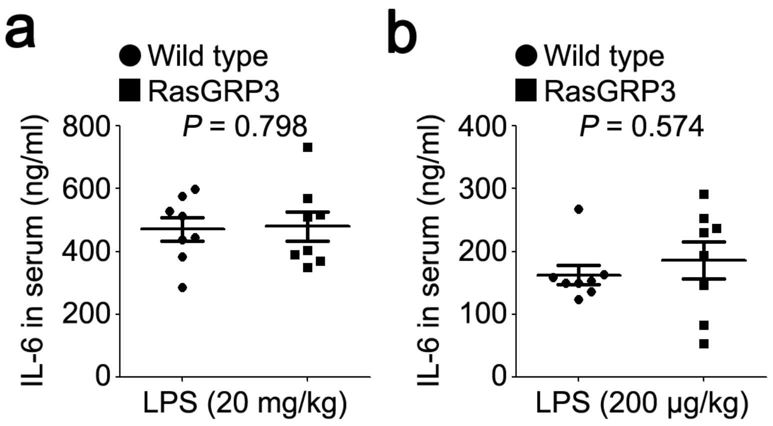

Supplementary Figure 10. IL-6 levels in the serum of wild type mice or RasGRP3 transgenic mice. Indicated mice were intraperitoneally injected with 20 mg/kg (**a**) or 200 μg/kg (**b**) LPS for 2.5h. IL-6 levels in serum were determined by ELISA. Data are presented as mean±s.d. of 8 mice per group (Mann-Whitney U test). Data are representative of three independent experiments.

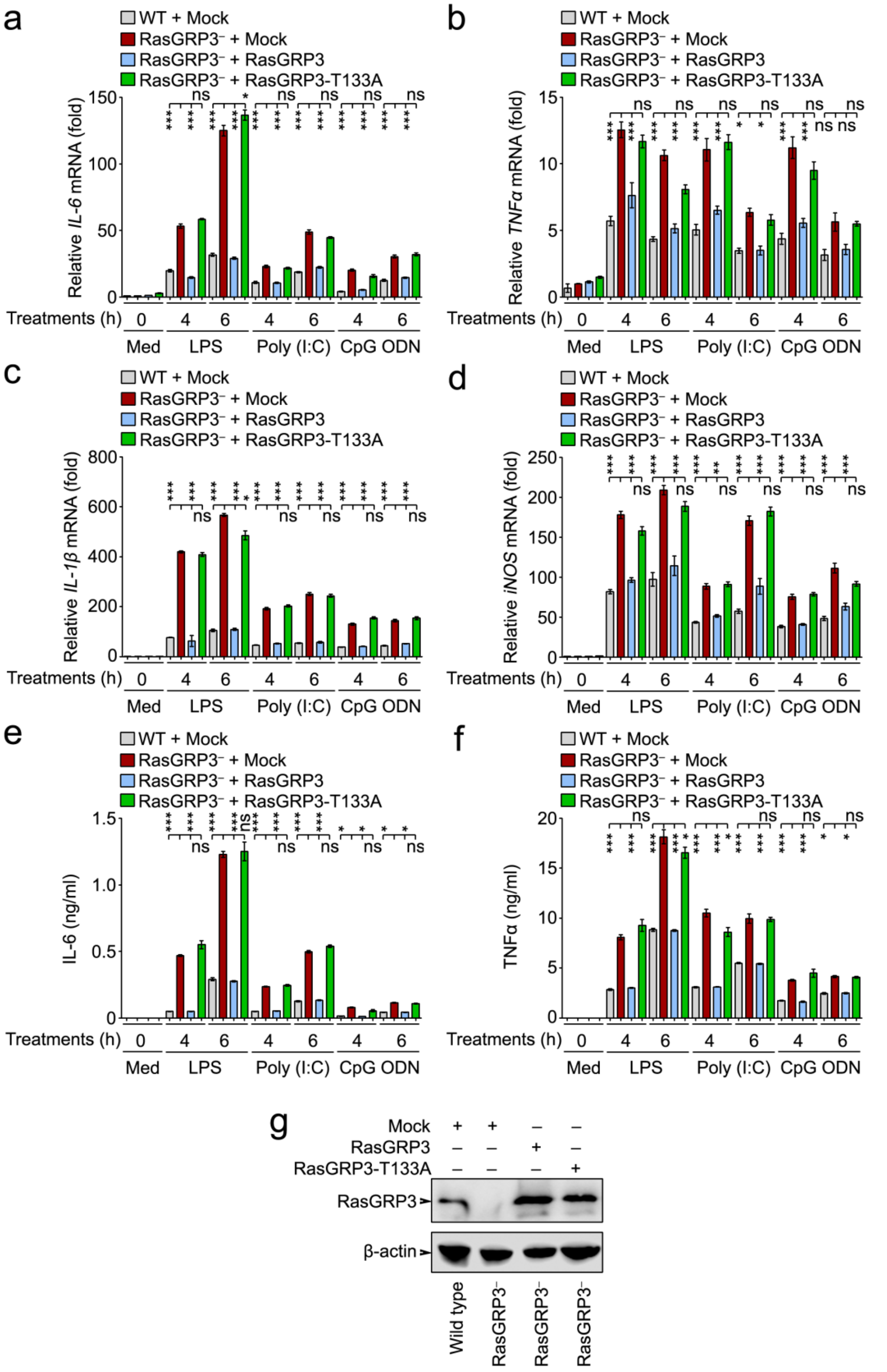

Supplementary Figure 11. Rescue experiments using RasGRP3 overexpression in RasGRP3-deficient RAW264.7 cells. (**a-f**) Wild type (WT) or RasGRP3⁻

RAW264.7 cells were transfected with indicated vectors. 48h later, cells were treated with or without (Med) 1 ng/ml LPS, 2.5 μg/ml Poly (I:C) or 0.5 μM CpG ODN as indicated. Then mRNA levels of indicated factors were determined by Q-PCR (**a-d**). IL-6 and TNFα in the culture supernatants were measured by ELISA (**e**, **f**). Data are presented as mean±s.d of triplicate samples. ns, not significant; *, $P < 0.05$; **, $P < 0.01$; ***, $P < 0.001$ (ANOVA). (**g**) Western blot assays of RasGRP3 (mutant) overexpression. Data are representative of three independent experiments.

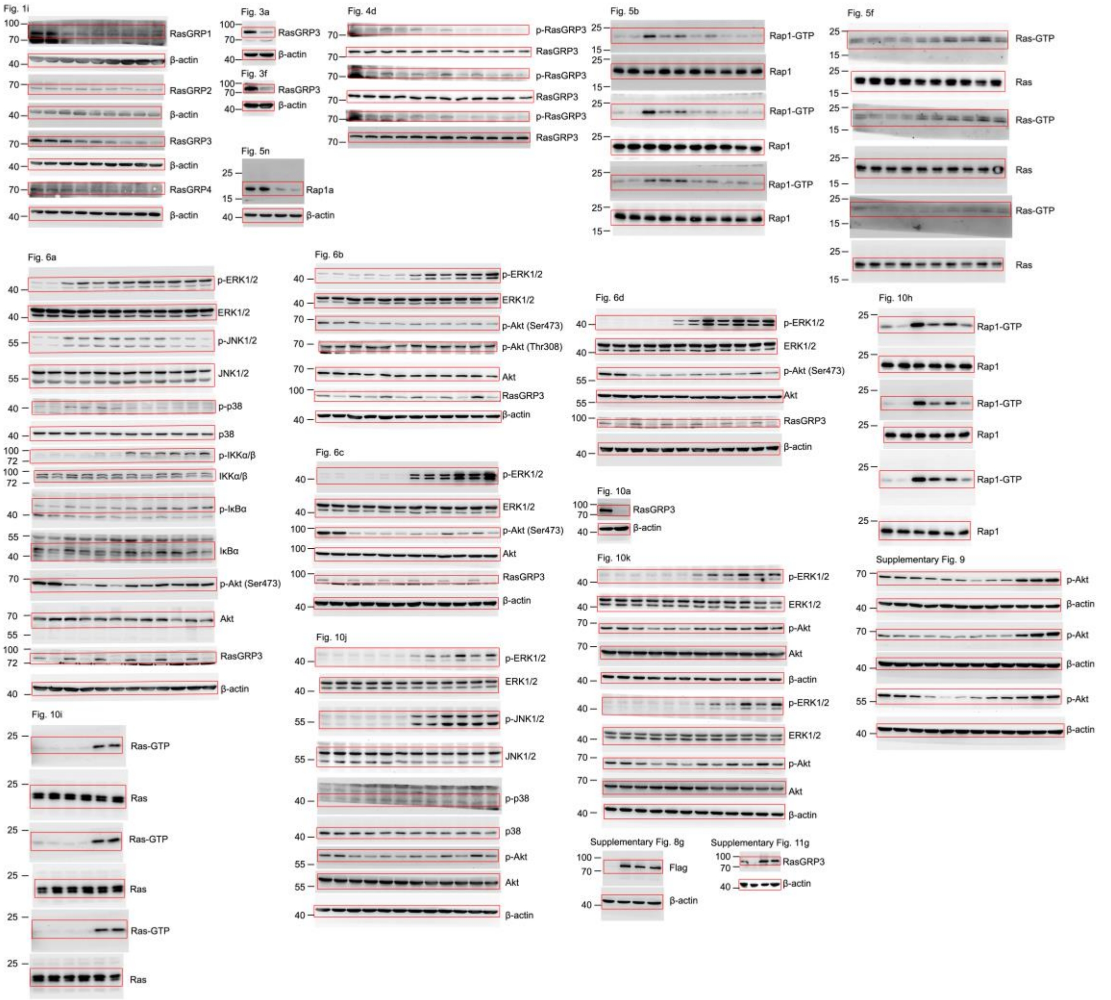

Supplementary Figure 12. Uncropped images of blots presented in the main paper. Molecular weight markers are indicated in kDa.

Supplementary Table 1. Primers used in vector construction.

Vector	Forward Primer (5'→3')	Reverse Primer (5'→3')
Mouse RasGRP3-Flag	GGCCTCGAGATGGATTATAAGGCATTCGACAACTTGGGGTCCAACGGGCTCGGGAAAGC	GTGGAATTCTCAGCCATCCTCACCACCCTGCTTGGCT
Mouse RasGRP3-T133A-Flag	GACTGGATGAGGAGAGTCGCACAGAGGAAAAAAGTAT	GATACTTTTTTCCTCTGTGCGACTCTCCTCATCCAGT
Human RasGRP3-Flag	GGCCTCGAGGCCATGATGGACTACAAGGATGACGATGACAAGGGATCAAGTGGCCTTGG	GTGGAATTCCACAAGCCAACATTATTGCCTTCAGTT
Human RasGRP3-T133A-Flag	ACTGGATGAGAAGAGTCGCACAGAGGAAAAAAGT	ACTTTTTTCCTCTGTGCGACTCTTCTCATCCAGT
Human RasGRP3-G558R-Flag	CTTGAGCAGTGGTCATAGGTCACTGCCTGGAAG	CTTCCAGGCAGTGACCTATGACCACTGCTCAAG

Supplementary Table 2. Primers used in Q-PCR assays.

Gene Target	Forward Primer (5'→3')	Reverse Primer (5'→3')
Mouse *RasGRP1*	ACTGCCACCTCATCGACAC	CACTTTGCGCTTCTTGCTAGTA
Mouse *RasGRP2*	GCTCCGTGGTTGCATCGAA	CTGTGGATCTCGCACCTTTCC
Mouse *RasGRP3*	GGGAAAGCGGCAACACTAGAT	GGGCAAGTAACTGTCGTTCAG
Mouse *RasGRP4*	TGGCGTCCATGAATCTGGG	CGGATACATTCCTCCAATAGCTC
Mouse *Rap1a*	CAGGAACCGAGCAATTTACAGC	TGTTCTTTGCCAACTACCCGT
Mouse *IL-6*	GAGTTGTGCAATGGCAATTCTG	GCAAGTGCATCATCGTTGTTCAT
Mouse *TNFα*	CCCTCACACTCAGATCATCTTCT	GCTACGACGTGGGCTACAG
Mouse *IL-1β*	GAAATGCCACCTTTTGACAGTG	CTGGATGCTCTCATCAGGACA
Mouse *iNOS*	ACATCGACCCGTCCACAGTAT	CAGAGGGGTAGGCTTGTCTC
Mouse *β-actin*	AGTGTGACGTTGACATCCGT	GCAGCTCAGTAACAGTCCGC
Human *RasGRP1*	CAGGAACTGGTGAAAGCTAAGG	AGTCACGGGCATTGATTTGAG
Human *RasGRP2*	GAGCATCTCACCTACTTGGAGT	AGCCATGAGTCACGAAACTGT
Human *RasGRP3*	GGGAAAAGCCTGTCTGCTGTT	GCTCCAGAAAAGTGAGGTGCT
Human *RasGRP4*	GTACTGGCTGATGCGACACC	GGCCCAGAAACGACCTATGAC
Human *IL-6*	ACTCACCTCTTCAGAACGAATTG	CCATCTTTGGAAGGTTCAGGTTG
Human *TNFα*	CCTCTCTCTAATCAGCCCTCTG	GAGGACCTGGGAGTAGATGAG
Human *IL-1β*	AGCTACGAATCTCCGACCAC	CGTTATCCCATGTGTCGAAGAA
Human *iNOS*	TTCAGTATCACAACCTCAGCAAG	TGGACCTGCAAGTTAAAATCCC
Human *β-actin*	CATGTACGTTGCTATCCAGGC	CTCCTTAATGTCACGCACGAT

Supplementary Table 3. siRNA sequences used in this study.

siRNA Target	Sense (5'→3')	Antisense (5'→3')
Mouse RasGRP1	GCUCCAUCUAUUCCAAGCUTT	AGCUUGGAAUAGAUGGAGCTT
Mouse RasGRP2	GGGCCUUAAAUGUAGAGCUTT	AGCUCUACAUUUAAGGCCCTT
Mouse RasGRP3	GAGUCUGUGUUUCGAAACUTT	AGUUUCGAAACACAGACUCTT
Mouse RasGRP4	CACCUACCCAAGCUGAAUATT	UAUUCAGCUUGGGUAGGUGTT
Mouse Rap1a	GACCUGGUCAGACAGAUAATT	UUAUCUGUCUGACCAGGUCTT
Mouse Rap1b	GUGCGGCAAAUUAACAGAATT	UUCUGUUAAUUUGCCGCACTT
Human RasGRP1	GCGGGAUGAACUGUCACAATT	UUGUGACAGUUCAUCCCGCTT
Human RasGRP2	GCAUGGGCUUCGUACACAATT	UUGUGUACGAAGCCCAUGCTT
Human RasGRP3	GGAUCUCAUUCACUGAUUATT	UAAUCAGUGAAUGAGAUCCTT
Human RasGRP4	GGACCUCUUCUACACGGAATT	UUCCGUGUAGAAGAGGUCCTT
Control siRNA	UUCUCCGAACGUGUCACGUTT	ACGUGACACGUUCGGAGAATT
